# Variable progressive behavior of *Klebsiella pneumoniae* at different sites of infection

**DOI:** 10.3389/fimmu.2026.1775450

**Published:** 2026-04-13

**Authors:** Tarek A. Ahmad, Dina M. Tawfik, Hossam-Eldine M. Ghoneim, Manal A. Nabil, EL-Sayed H. El-Ashry, Laila H. El-Sayed

**Affiliations:** 1 SeptivaK Research Group, Department of Immunology and Allergy, Medical Research Institute, Alexandria University, Alexandria, Egypt; 2 Library Sector, Bibliotheca Alexandrina, Alexandria, Egypt; 3 Department of Immunology and Allergy, Medical Research Institute, Alexandria University, Alexandria, Egypt; 4 Department of Chemistry, Faculty of Science, Alexandria University, Alexandria, Egypt

**Keywords:** antigenic determinants, diagnostic microbiology, epitopes’ mapping, host-pathogen interaction, *Klebsiella pneumoniae*, vaccine design

## Abstract

*Klebsiella pneumoniae* has persisted for decades as a major global health threat. Although vaccination is widely recognized as the most effective preventive strategy, no vaccine has been commercialized despite more than 50 years of intensive research. While numerous computational studies have screened epitopes from over 25 different *Klebsiella* protein antigens, none have progressed to vaccine development, underscoring the urgent need for innovative approaches to define the immunological signatures of this pathogen. In this study, infections caused by classical *K. pneumoniae* (cKp) at distinct anatomical sites were experimentally mimicked in an animal model to investigate antigen-specific host immune responses during infection. Humoral immune responses against major protein and polysaccharide antigens were characterized using immunoblotting and quantitative ELISA, while cellular immune activation was evaluated through lymphocyte proliferation assays. The results revealed the site-specific and progressive antigenic expression patterns of *K. pneumoniae*, reflecting dynamic host–pathogen interactions across different infection models. Notably, immunoglobulin profiling demonstrated strong potential to discriminate both the stage and route of *K. pneumoniae* infection. These findings underscore the importance of polysaccharide antigens and protein target mapping prior to *in silico* epitope prediction for vaccine and diagnostic production.

## 1 Background

*Klebsiella pneumoniae* is one of the most challenging community-acquired and nosocomial bacterial pathogens worldwide. It is the main cause of several human and animal diseases collectively called *Klebsiella*-associated syndromes (1). As an intestinal tract commensal, classical *K. pneumoniae* (*cKp*) acquired multidrug resistance toward known therapies (2–4), as confirmed in bibliometric large-scale studies (5). The development of hypervirulent (*hvKp*), motile strains of *Klebsiella* (6–8) and metastatic infection induce high mortality rates (9). Therefore, prevention by prophylactic immune preparations flared in the dark and was followed by different research schools within the last 50 years (10); however, none reached the market as of yet (11, 12). The management of the significant challenge for the all-time emerging pathogen *K. pneumoniae* is still a priority (13, 14).

Although several research highlighted the importance of epitope mapping to design powerful vaccines and sensitive diagnostics (15, 16), very few attempts for epitope mapped vaccine against *K. pneumoniae* reached the evaluation stages, but they have not progress into a commercial vaccine or diagnostic (17, 18). On the other hand, only two molecular attempts were proposed to investigate the immune response against the main protein (19) and protein/polysaccharide (20) antigenic determinants in the sera of infected human patients. From 2015 until the present, a reasonable plethora of computer-based epitopes were proposed by different researchers around the world against the pathogen’s short protein immunomes in the peptidoglycan-associated lipoprotein, ribonuclease, and synthetase enzymes, type I fimbriae adhesin antigens (Fim: A, F, G and H), as well as proteins that mediate capsular polysaccharide (CPS) biosynthesis, together with different transporters that showed an exceptional preference among researchers such as outer membrane proteins (OMPs: A, K35/F, K36/C, K17/X, N and K22/W), ferric (FepA) and ferrous (FepB) enterobactin siderophores, TonB-dependent siderophore receptor, zinc transporter (ZnuA), cation efflux transporter for silver and copper (CusC), the phosphate starvation porin (PhoE) (21–25), and pangenome screening for protein epitopes (14, 26–29). However, this vast array of computational and molecular research never fulfilled the unmet need to circulate a new vaccine into the pipelines to prevent *K. pneumoniae.*

At this stage of research and more than ever before, there is a clear necessity to reorient the trend of studies and to identify the current research knowledge gap (14, 30). Beyond epitope mapping, the concept of deciphering the “molecular signature of immunity” was shown to be crucial in designing vaccines and diagnostics for pathogens with complex interactions, such as bacteria (31). Simultaneously, a corner stone of designing vaccines or diagnostics is to know the timing and potency of the immune system response to a particular antigen, especially that research highlighted the variable metabolomic behavior of pathogens (32) that can be recognized by the immune system.

We hypothesized that the major polysaccharide (CPS and LPS) and protein (OMPs and FIM) antigens of *Klebsiella pneumoniae* exhibit dynamic, site-specific, and stage-dependent immunological profiles during infection. Accordingly, the aim of this study was to systematically characterize the antigen-specific immunological signatures elicited by these determinants through the quantitative assessment of both humoral and cellular responses in controlled animal models of the bloodstream, urinary tract, and respiratory tract infections. We further postulated that this *in vivo* and cost-effective framework would establish a foundational platform for subsequent epitope-mapping investigations.

## 2 Materials and methods

### 2.1 Experimental animals, ethics, and bacterial isolate

White New Zealand rabbits were used as the experimental infection model as they have been previously proven to be successful models to study different infections by *cKp* (33–35). Healthy mixed-sex not-previously-immunized 45-day-old rabbits weighing 1.5 ± 0.1 kg were purchased from the farm of the Faculty of Agriculture in Alexandria University. The animals were kept in the housing facility of the Medical Research Institute (MRI) of Alexandria University in a separate ventilated ward containing cages of appropriate space, which were continuously cleaned and sanitized. The animals were continuously supplemented with fresh water, fed with high-grade specialized feed for rabbits, supplemented with essential minerals and vitamins for healthy growth, and kept under daily veterinary inspection of the MRI animal house facility. The animals were left to climatize under medical observation for two weeks before the experiments were started at the age of 60 days. Given that klebsiellosis was not reported in rabbits older than 60 days, the infection would initiate immunity until cleared by the immune system without harming the animals (36). All experiments were conducted under the ethical conditions’ codes of the university. The animals were numbered, divided into groups of seven for each experimental model, and housed individually in their cages. The *K. pneumoniae* serotype K41:O1 purchased from Institute Pasteur Collection encoded CIP53.16 (provided by the first author) was grown on brain heart infusion broth overnight under standard conditions. This *cKp* strain was used, as the CPS is not overexpressed (*hvKp*) nor mask the other antigens studied (37). Freshly prepared whole cells were washed and diluted in phosphate-buffered saline (PBS) to a final concentration of 2 × 10^5^ CFU/mL from a starting suspension of 2.4 × 10^8^ CFU/mL (OD_600_ = 0.5), according to the McFarland standard method (38).

### 2.2 Experimental models

In principle, the mixed-sex rabbits were divided into three groups each of seven and inoculated with a sublethal dose of 70 µL of the stock solution of the living bacterial cells (39). Group 1, for the sepsis-model (SP), was injected intraperitoneally. Group 2, the respiratory-model (RT), was slowly inoculated endotracheally (half-way mucosal) using a blunt-tipped needle. In group 3, for the urinary tract model (UT), the female rabbits were left overnight with no water (to minimize washing the dose by urine), then inseminated in the morning via the trans-urethral route, and kept in a gentle inverted cradle position for 20 min. Female rabbits were used in this model to facilitate the trans-urethral inoculation procedure and ensure technical consistency. Two rabbits for each experimental group were inoculated with sterile phosphate-buffered saline according to the attributed route of infection as control references.

### 2.3 Immunization schedule and blood sampling

**Figure 1:**
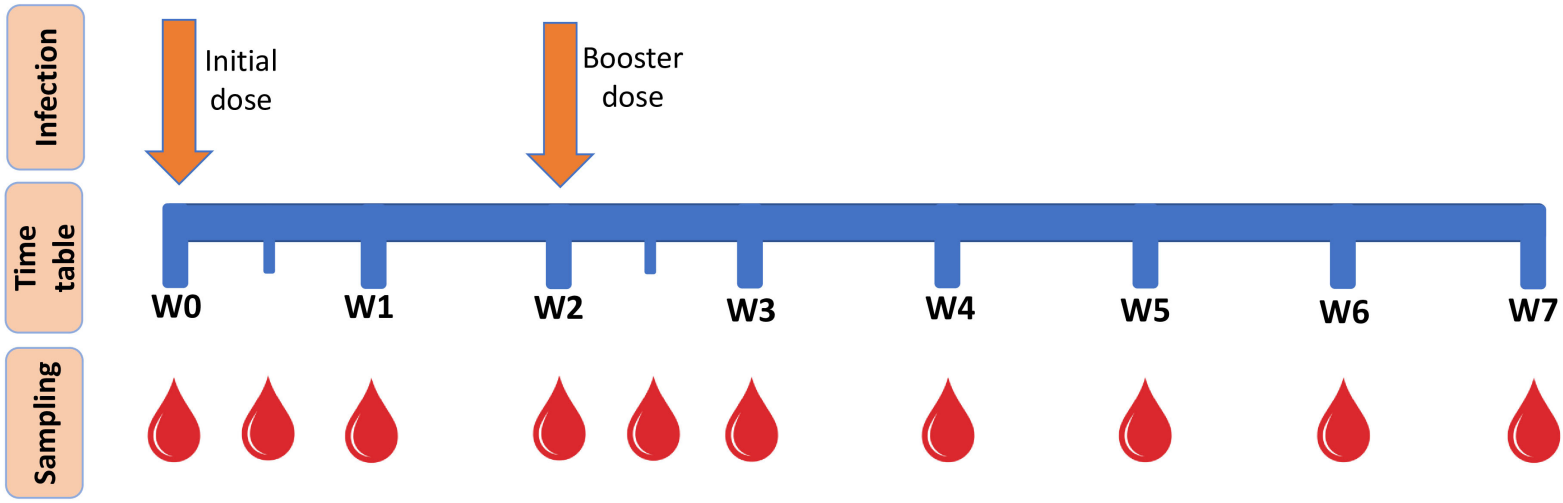
Time schedule for the infection of the animal models by *K. pneumoniae* doses and collection of blood samples. W(n) is number of weeks elapsed since the initial infection. The three experimental animal models were inoculated at W(0) and W(2). Blood samples were collected on a weekly basis; however, following each inoculation, an additional sample was collected once at the mid-week point. The blood samples were processed according to the further required cellular or humoral investigation.

The rabbits were inoculated twice at a 2-week interval (initial infection and re-infection booster dose). Blood samples were collected from the marginal ear vein of the rabbits in heparinized and serum vacutainers weekly, with an additional single collection performed at the mid-week point following each inoculation (**Figure 1**). The sera were separated from the collected blood samples and kept at -80 °C for further evaluation. The lymphocytes were separated from the collected fresh whole blood sample by using Ficoll (1.083 g/mL; Sigma-Aldrich, USA) gradient separation for immediate testing (40).

### 2.4 Major antigen purification, quantification, and purity assessment

Pure antigens were prepared to enable the detection of low copies of epitopes that could be hindered by more dominant ones, regardless of their immunogenicity (41). Lipopolysaccharide (LPS), outer membrane proteins (OMP), and bacterial fimbrins (FIM) were extracted from the CPS-reduced *K. pneumoniae* cells grown under standard conditions (17) until the mid-log phase using the methods of Westphal and Jann (42), Filip et al. (43), and Witkowska et al. (44), respectively. The FIM extract was further purified using Sephadex G-50 (Sigma-Aldrich, USA). The CPS was extracted from bacterial cells grown overnight in the late log-phase under standard conditions using 0.1% hexadecyltrimethylammonium bromide (Cetavlon)/ethanol precipitation method (45). The identity and purity of the different antigens were examined by electrophoresis on 12% polyacrylamide gels (PAGE) using Laemmli buffer system (46) concurrently with a standard protein marker (10–170 kDa; Nippon Genetics, Japan) (**Supplementary 1**). The gels were stained by using Alcian blue stain (Serva, Germany) to visualize the acidic CPS (47) and by using silver stain to identify both the polysaccharides and the protein bands (48). The identified quality-controlled pure antigens were preserved in buffer solutions at -80°C until further use. The proteins were quantified by spectrophotometric methods against a standard serial dilution (49), while the polysaccharide concentration was determined by the Dubois method (50).

### 2.5 Western blot technique

The qualitative humoral immune response was studied by using the immunoblotting technique, due to its advantages in screening polysaccharide antigens, as well as both linear and conformational proteins (15). The standard whole *K. pneumoniae* cells were subjected to electrophoresis on 12% polyacrylamide gels using the Laemmli buffer system (46) versus a standard protein marker 10–170 kDa (Nippon, Japan). The antigens were transferred onto 0.2-µm-pore-size nitrocellulose membrane using the method of Towbin et al. (51) and modified by using the method of Porsch-Ozcurumez et al. (52). Two membrane-transferred lanes were incubated with the tested rabbit antisera as primary antibody and then goat anti-rabbit whole immunoglobulin–horse radish peroxidase conjugate (Ig-HRP, from Serotec GmbH, Germany) as secondary antibody according to the manufacturer’s procedure.

### 2.6 Evaluation of the humoral immune response

ELISA was used to quantify the B-cell humoral immune response (15). In brief, ELISA plates were coated according to Huang et al. (53), with an optimized concentration of the purified antigens (LPS, OMPs, FIM, and CPS) as well as the formalin-inactivated whole bacterial cells (FIWC) in each of the three replicas. The antigens’ concentrations were quantified using the method of Dubois et al. (50) and using the UV-monitored standard curve of bovine serum albumin (BSA) at 280 nm. The different tested antisera were used as primary antibody, while commercial (Serotec GmbH, Germany) antisera against rabbit IgA, IgG, and IgM conjugated to HRP were used as secondary antibody to detect the specific immunoglobulin response. The color developed by the substrate was measured by using a spectrophotometer (Infinite F50 Tecan, Switzerland) at 450 nm, noting that the control and blank wells were settled to lack the primary antibody and the coating antigens, respectively.

### 2.7 Lymphocyte proliferation assay

The proliferation assay was used to measure the cellular immune response (16). The viability of the previously isolated lymphocytes was performed using the trypan blue method, and cell counts were adjusted to 5 × 10^5^ cell/mL in complete RPMI-1640 medium (Biowest, France) for culturing. To assess the immune proliferation, neutral red dye media was prepared in a concentration of 40 µg/mL in complete RPMI-1640 media and incubated with three replicas of the centrifuged cells at the same conditions for 2 h. The neutral red media was centrifuged to remove any insoluble suspension. Cell proliferation was measured at 540 nm absorbance (54).

### 2.8 Statistical analysis of the data

The data of the ELISA and proliferation assays were processed using SSPS and analyzed by using independent samples *t*-test. The ELISA data were visualized as mean fold increase, and the proliferation assays’ data were presented as stimulation index (SI). The standard error (SE) and *p*-value were calculated on corrected optical density (OD). Statistical significance was determined at *p*-value ≤0.05.

## 3 Results

### 3.1 Antigens’ identity and purity

The silver staining of the purified LPS antigen did not show contaminating sharp bands of proteins. The smear confirmed the structural identity of the LPS as a low molecular weight lipid, being a densely stained smear, followed by the core antigen and minor amounts of O-antigen in the overnight grown bacteria tested. Moreover, the smear lacked CPS that appears as high molecular weight smear or acidic polysaccharides that respond to Alcian blue stain (**Supplementary 1**). On the other hand, the extracted CPS run on the PAGE appeared as a high molecular weight smear at the top of the gel that was both fully stained by the silver stain and Alcian blue, confirming the identity of the acidic CPS while not showing any sharp band for contaminating proteins. Simultaneously, the silver staining of the purified OMP preparation run on PAGE did not show any smear background for any type of polysaccharide contaminants while displaying the typical pattern of OMP of iron-replete *K. pneumoniae*. The bands showed at molecular weights of 15, 17, 20, 32, 40, and 60 kDa. The bands’ intensity was approximately equivalent, with a rational distinctive increased intensity of the 17-, 20-, 32-, and 40-kDa bands. The silver staining of the purified FIM preparation run on PAGE did not show any smear background for polysaccharide impurities while displaying sharp protein bands at molecular weights of 11–13 and 28.8 kDa. The higher molecular weight band of FIM was expressed in a remarkably much denser intensity.

### 3.2 Deciphering the humoral immune response

The results of the western blot were studied by a visual comparison of the positions of the developed bands of the bacterial immunogens. The results confirmed the presence of an antibody immune response against the four main antigens studied in that article. The western blot data showed that the OMPs at molecular weights of 60, 40, 32, 20, and 15 kDa were able to elicit a clear immune response starting from day 3 (**Supplementary 2**) and along the experiment until day 49. Simultaneously, a prominent distinctive immune response clearly appeared starting from day 16 and onward against the ladder-like structure of LPS in the SP-model. However, the antibody response against the FIM band at a molecular weight of 28.8 kDa was visible at the third day post-initial infection of the sample in the mucosal models; then, the response disappeared 1 week later, whereas the CPS showed a very faint response that can be barely detected by the western blot throughout the whole experiment. Most probably, this is due to the poor transferability of the high molecular weight CPS to the nitrocellulose membrane sheets during the western blot process.

**Figure 2:**
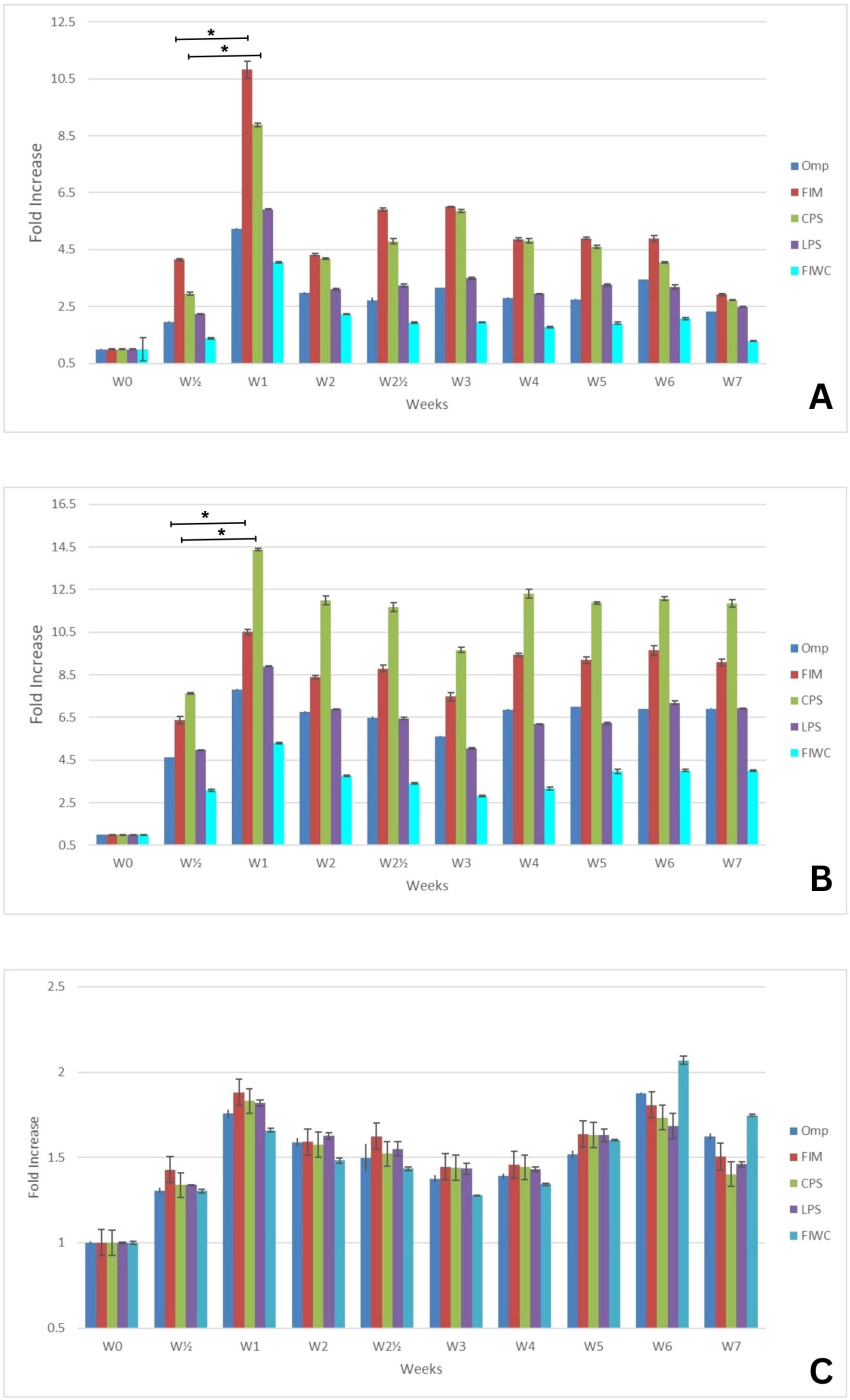
Mean fold increase of **(A)** IgM, **(B)** IgA, and **(C)** IgG antibody titers against the four tested antigens in the urinary tract infection model (UT) over a 7-week period (W0–W7). The initial infection was induced at week 0 (W0), and a booster (reinfection) dose was administered at week 2 (W2). Data are presented as mean fold increase. The corrected optical density (OD) values were statistically analyzed to calculate the standard error (SE) and to determine *p*-values at intervals of each individual sample or every two consecutive samples. Key: The four tested antigens are outer membrane proteins (Omp), fimbrial proteins (Fim), capsular polysaccharides (CPS), lipopolysaccharides (LPS), and formalin-inactivated whole bacterial cells (FIWC). The number (*n*) of weeks elapsed since the initial infection are presented as W(n), and the symbol (*) denotes the highest fold increase with statistical significance (*p* ≤ 0.05).

**Figure 3:**
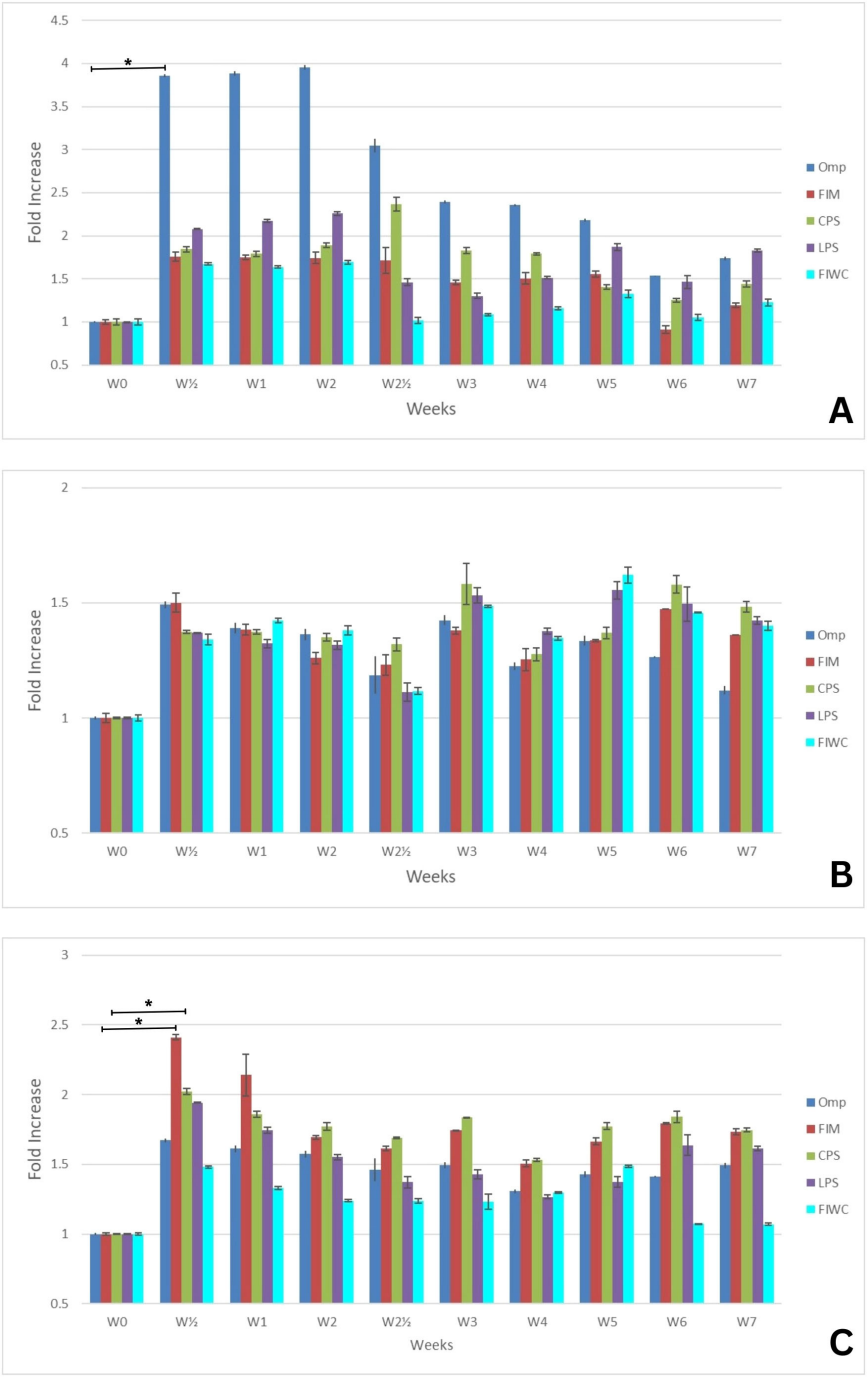
Mean fold increase of **(A)** IgM, **(B)** IgA, and **(C)** IgG antibody titers against the four tested antigens in the respiratory tract infection model (RT) over a 7-week period (W0–W7). The initial infection was induced at week 0 (W0), and a booster (reinfection) dose was administered at week 2 (W2). Data are presented as mean fold increase. The corrected optical density (OD) values were statistically analyzed to calculate the standard error (SE) and to determine *p*-values at intervals of each individual sample or every two consecutive samples. Key: The four tested antigens are outer membrane proteins (Omp), fimbrial proteins (Fim), capsular polysaccharides (CPS), lipopolysaccharides (LPS), and formalin-inactivated whole bacterial cells (FIWC). The number (*n*) of weeks elapsed since the initial infection are presented as W(n), and the symbol (*) denotes the highest fold increase with statistical significance (*p* ≤ 0.05).

**Figure 4:**
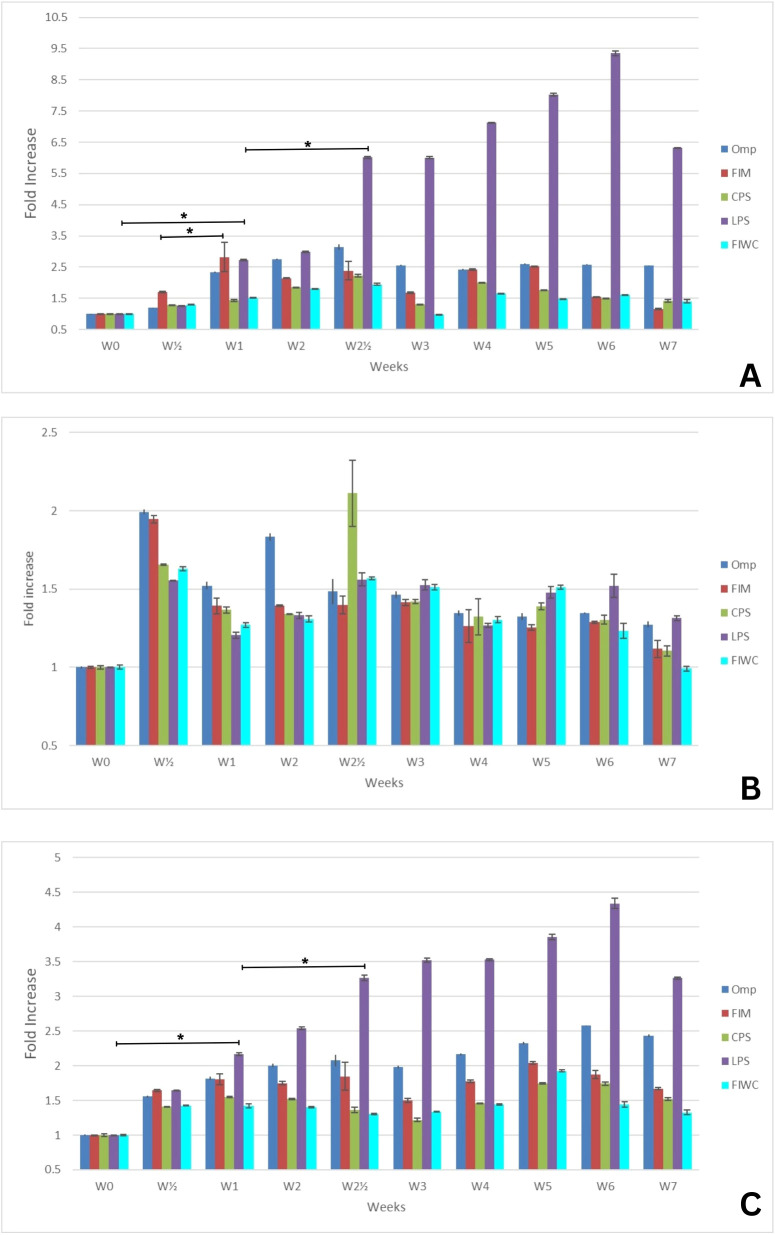
Mean fold increase of **(A)** IgM, **(B)** IgA, and **(C)** IgG antibody titers against the four tested antigens in the sepsis infection model (SP) over a 7-week period (W0–W7). The initial infection was induced at week 0 (W0), and a booster (reinfection) dose was administered at week 2 (W2). Data are presented as mean fold increase. The corrected optical density (OD) values were statistically analyzed to calculate the standard error (SE) and to determine *p*-values at intervals of each individual sample or every two consecutive samples. Key: The four tested antigens are outer membrane proteins (Omp), fimbrial proteins (Fim), capsular polysaccharides (CPS), lipopolysaccharides (LPS), and formalin-inactivated whole bacterial cells (FIWC). The number (*n*) of weeks elapsed since the initial infection are presented as W(n), and the symbol (*) denotes the highest fold increase with statistical significance (*p* ≤ 0.05).

The correlation between the semi-quantitative but mostly qualitative data of the western blot and the data obtained from the quantitative ELISA experiments showed the time-related patterns of antibodies’ response against the different pure antigens. In principle, the generated immunoglobulins were quantified in the sera of the different animal models against each tested antigen by ELISA, and the absorbance was read at 450 nm. **Figures 2**–**4** present the measurements of immunoglobulins (IgA, IgM, and IgG) elicited against the four selected antigens across the three different infection models of *K. pneumoniae*. The antibody responses are expressed as fold-rise in antibody titers. Data are presented in the graphs as mean values ± standard error (SE). Statistical analyses were performed to determine the significance levels, which are provided in the **Supplementary Files**. Unfortunately, the sensitivity provided by the immunoblot gave suboptimal comparable information for the components of each antigen. The method did not succeed to point overlapping detections of antigens, and the results lost consistency with the progressive development of more antigen-antibody reactions (**Supplementary 2**).

As shown in **Figure 2**, for the urinary tract (UT) infection model, the host showed variable interactions toward the different applied antigens. The titer of IgM and IgA (**Figures 2A, B**) significantly raised (*p* ≤ 0.05) from the third day post-initial infection toward all tested pure antigens. The generated IgM (**Figure 2A**) displayed the highest titer toward FIM, followed by CPS, that almost reached 10-fold at 1 week post-initial infection, and then the titer gradually decreased regardless of the booster dose. Simultaneously, the IgA titer (**Figure 2B**) showed a clear superior increase at 1 week post-initial infection against CPS that reached 14-fold, followed by FIM, denoting the overexpression of those two antigens together in the early stages of the urinary tract infection by the pathogen. The IgA titer level showed a remarkably significant protective increase (fourfold) in antibody production against all tested antigens that was retained along the 7 weeks of the experiment and was not affected by the booster dose, confirming the role of mucosal immunity in the urinary tract. Although the IgG titer increased against almost all tested antigens, the titer (**Figure 2C**) was significantly lower (~1.5–2 fold) than that of IgM and IgA, and it did not show a remarkable increase after the booster infection.

As shown in **Figure 3**, for the endotracheal respiratory (RT) infection model, the host showed a rapidly high (approximately fourfold) and significant (*p* ≤ 0.05) increase in IgM titer (**Figure 3A**) against OMP at 2 days post-initial infection, denoting the outstanding expression of the OMP at the early stages of respiratory infection by the pathogen. This titer was stable until it started to decline gradually in the second week post-infection regardless of the booster infection. The IgA titer (**Figure 3B**) increase showed an irregular pattern against all tested antigens and never trespassed 1.5-fold increase. On the other hand, the IgG immune response (**Figure 3C**) against the endotracheal infection showed the correlated dominance of the antibodies against CPS and FIM that lasted until the end of the seventh week. The booster infection at the end of the second week did not boost the titer of the antibodies against any tested antigen, while IgG and IgM generated against the formalin killed whole cell (FIWC) of *K. pneumoniae* manifested the lowest titer increase.

As shown in **Figure 4** of the intraperitoneal sepsis (SP) model of infection, the host manifested a delayed high increase in IgM titer (**Figure 4A**) after the third day of infection for all tested antigens. Unlike the other infection models, the outset remarkable increase in IgM titer was seen against LPS, which was affected by the booster dose of infection at week 2, but with a significantly faster response more than that induced by the initial dose. This highlights the special initial immune response against that structural antigen that was always produced at the same amount during infection in all models. However, the IgM titer against LPS started to decrease at 6 weeks post-initial infection, while the IgM response against the other tested antigens did not show a higher increase in titer beyond 2.5-fold, with a slight superiority of OMP. The pattern of IgA response (**Figure 4B**) toward the infection showed an irregular pattern against most tested antigens. On the other hand, the infection by a sublethal dose of living cell elicited an IgG response (**Figure 4C**) with a remarkably (~4.5-fold) significant increase in titer against LPS that was affected by the infection booster dose and progressed until the sixth week. Except for OMP and LPS, the generated IgG against all other tested antigens showed a protective increase of twofold starting from the first week post-initial infection, was not affected by the booster dose of infection at week 2, and persisted until the seventh week.

The immune response of IgM and IgG showed a significant protective titer in all infection models. As shown in **Figure 2B**, the IgA showed a predominant titer increase in the UT-model in comparison with the SP-model and RT-model (**Figures 3B** and **4B**). Although the different classes of tested antibodies originated from the infection by the living whole cells, the overall antibody back-response against the FIWC was the least in comparison to the individually purified antigens. This is probably due to the steric hindrance of antigens in the whole and intact undigested living cell or to the lower abundance level of antigens in the FIWC in comparison to the individually purified ones.

### 3.3 Lymphocytic proliferation assay

**Figure 5:**
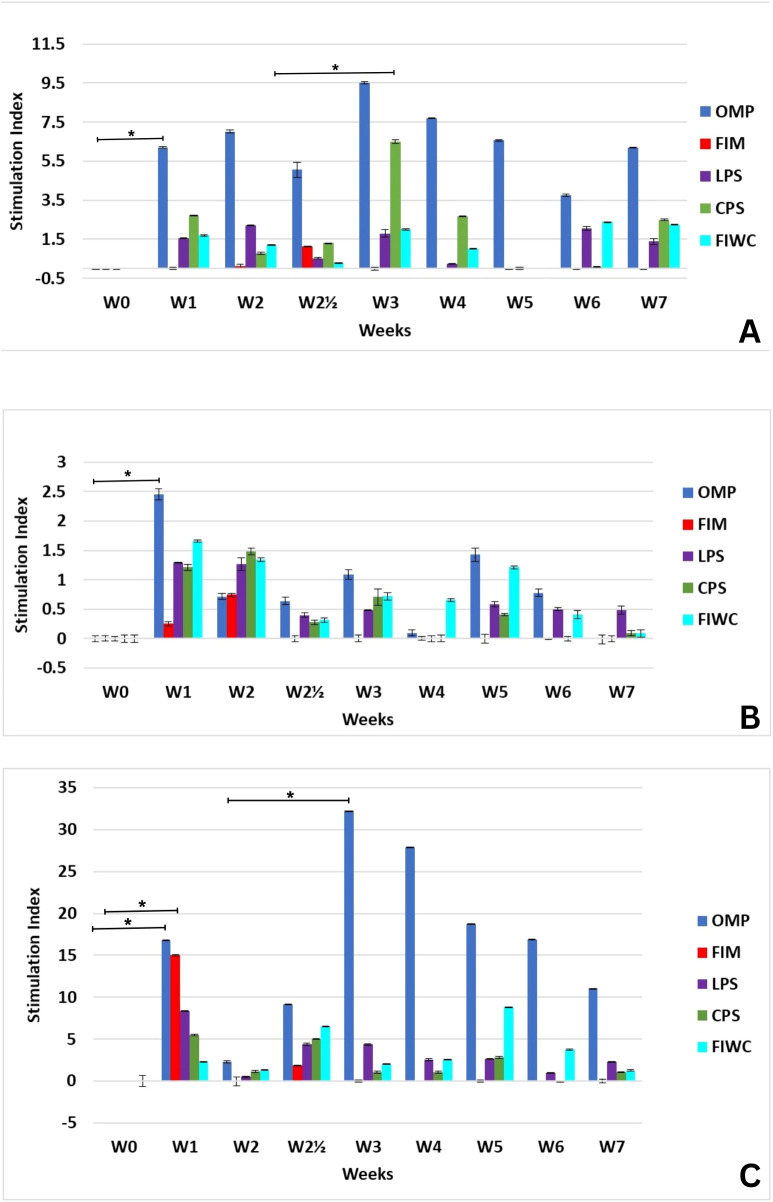
Mean of the stimulation index (SI) of the lymphocytes induced by the four tested antigens over a 7-week period (W0–W7) at the **(A)** urinary tract infection, **(B)** respiratory tract, and **(C)** sepsis models. The initial infection was induced at week 0 (W0), and a booster (reinfection) dose was administered at week 2 (W2). Data are presented as mean stimulation index. The corrected optical density (OD) values were statistically analyzed to calculate the standard error (SE) and to determine *p*-values at the intervals of each individual sample or every two consecutive samples. Key: The four tested antigens are outer membrane proteins (Omp), fimbrial proteins (Fim), capsular polysaccharides (CPS), lipopolysaccharides (LPS), and formalin-inactivated whole bacterial cells (FIWC). The number (*n*) of weeks elapsed since the initial infection are presented as W(n), and the symbol (*) denotes the highest SI increase with statistical significance (*p* ≤ 0.05).

Fundamentally, the proliferation assay was implemented by measuring the absorbance of the remaining neutral red dye after its uptake by proliferating lymphocytes as a response to the stimulation induced by each of the tested antigens. The readings for the blank and the non-stimulated cell samples were deducted from the absorbance readings. Given that, concanavalin A was applied as a positive control. To facilitate the data interpretation of the proliferation assay, the stimulation index (SI) was calculated as being the net division value of the stimulated cell supernatant absorbance divided by non-stimulated cell supernatant absorbance. As displayed in (**Figure 5A**), the OMP showed an absolute dominance to induce cellular proliferation as early as the third day post-initial infection in the UT-model. The stimulation index reached 7.2 times after the initial dose and then increased to 9.7 times after the booster dose at the middle of the third week post-initial infection. This stimulation remained significantly recognizable until the end of the experiment at the sixth week and retained a stimulation index of 6. CPS gave a similar behavior to that manifested toward OMP, but with a lower stimulation index that never exceeded 6.2 times at the middle of the third week post-initial immunization, whereas all other purified antigens did not give a significant increase in the stimulation index. However, FIWC gave a similar behavior to that of OMP and CPS, with a very low stimulation index. This may be due to the fact that FIWC is the parent component for OMP and CPS, while their individual concentration or potential display to the immune system in the FIWC is much lower than that found in their pure form.

In the RT-model (**Figure 5B**), OMP had a superior stimulation index over the other tested antigens that reached 2.5 times at 1 week post-initial infection. However, this stimulation did not improve further after the booster dose at week 2. In general, the endotracheal infection route had a relatively lower stimulation index for all types of antigens. However, the SP-model (**Figure 5C**) expressed the absolute highest stimulation index post-initial infection at the middle of the first week by OMP that reached 17 times. This stimulation significantly responded to the booster dose, increased to reach 33 times three days post-booster infection, and then gradually decreased to above 10 times during the sixth week post-initial infection. FIM initiated a lymphocytic activity that reached a stimulation index of 10 times at 3 days post-initial infection. However, this stimulation decreased thereafter and did not respond to the booster infection at week 2. In the SP-model, all types of purified or FIWC antigens manifested some kind of lymphocytic proliferation that reached a stimulation index of five times and responded to the booster dose.

## 4 Discussion

### 4.1 Interactions between host immunity and the pathogen

The administration of a sublethal bacterial dose to immunocompetent rabbits enabled the pathogen to proliferate and express its virulence factors *in situ* at the respective infection sites, provided that the rabbits were in health and age conditions that enabled them to withstand the infection while manifesting a complete immune response to clear the pathogen (36). The principal innovation of this study lies in mimicking *K. pneumoniae* infection at different anatomical sites within a suitable *in vivo* model while timely monitoring the host’s major immune responses along seven full weeks. Furthermore, the secondary infection administered at the end of the second week following the primary inoculation served both to challenge the developing primary immune response and to simulate the immunological effect of a booster dose using whole viable bacterial cells.

#### 4.1.1 Confirming facts

Evaluation of immune responses against antigens differing in type, nature, and site of exposure confirmed established immunological principles. LPS induced an early IgM and subsequent IgG response that was enhanced by the booster dose but declined after week 6 post-primary infection. This pattern reflects the exclusive B-cell-dependent response to LPS, a low–molecular weight polysaccharide hapten. Following the booster, the humoral response was more rapid due to pre-existing LPS-specific immune cells (55). Simultaneously, LPS elicited minimal cellular immunity, with only limited proliferation observed in the SP-model (**Figure 5C**), possibly due to weak T-cell involvement under conditions of high antigen exposure that were in close contact with the adipose tissue as will be discussed shortly (55). In contrast, the higher-molecular-weight CPS induced a partial T-cell–dependent response (55, 56) particularly evident in the UT-model and influenced by the booster dose (**Figure 5B**). Overall, protein antigens triggered a faster uptake and a more sustained humoral response than polysaccharides (**Figures 4** and **5**), as they are efficiently processed by antigen-presenting cells and promote long-term T-cell-mediated immunity rather than relying solely on short-lived B-cell responses (**Figure 5**) as well (55).

#### 4.1.2 Immunity at the mucosal barrier sites

All infection models inoculated with *K. pneumoniae* generated a protective IgM response against OMP. However, models representing mucosal barrier sites—namely, the urinary tract (UT; possibly vaginal) and respiratory tract (RT)—demonstrated a more rapid and pronounced IgM increase (greater than twofold within 3 days post-infection) compared with the SP-model (**Figures 2A**–**4A**). This reflects the nature of the respiratory and urinary tracts as external barrier sites that frequently face pathogens. They are equipped with opsonins, arsenals of resident dynamic antigen-presenting cells, and lymphatic tissue capable of quick antigen processing and immune response initiation (55, 57). The UT-model also exhibited a stronger cellular immune response against CPS than the RT-model, possibly due to a more efficient antigen presentation at the fully mucosal UT-site compared with the partially mucosal RT-site (57). Notably, this was the only model capable of inducing the protective levels of short-term, long-term, and mucosal antibodies (58). Induction of secretory immunity at the site of infection has the capacity to prevent the infection at the first line it harbors the host rather than curtailing the spread and infection (59). Collectively, these findings support that mucosal immunization triggers robust protective immune responses at the predominant sites of pathogen infection as reported in previous studies (57).

#### 4.1.3 Advantage of using pure antigens

Although immunity was initially induced using viable whole cells, the antibody response against formalin-inactivated whole cells (FIWC) was the lowest among the tested antigens (**Figures 2**–**5**). This may be explained by antigenic masking, whereby dominant surface determinants obscure less abundant but immunologically relevant epitopes during *in vitro* measurement. *In vivo*, however, pathogen processing by antigen-presenting cells exposes a broader range of antigenic determinants through intracellular degradation (55). Simultaneously, lymphocyte proliferation in response to FIWC was observed only in the SP-model and the UT-model (**Figures 5A, C**) that already showed increased proliferation to the individual composing antigens of the parent FIWC. Collectively, these findings suggest that applying purified antigens as vaccine or diagnostics production may offer some advantages over the whole cells in exposing the ready-to-deal-with immunomes to the APCs.

#### 4.1.4 Effect of antigens’ abundance and exposure

Our investigation further clarified the impact of antigen abundance and exposure on the quality of the immune response. Immunoblotting and antibody quantification demonstrated that the highly abundant secreted porins—OmpK32/A, OmpK36/C, OmpK17/X, and OmpK22/W (60)—elicited the most prominent antibody responses across all infection models. These findings are consistent with previous reports in sera from patients with *K. pneumoniae* infections (19, 20, 61), supporting the concept that the immune system preferentially targets externally exposed and abundantly expressed immunogens (62). Although some *in silico* mapping research screened for epitopes in those potent antigens, other ones screened for immunogens in less abundant ones (63). However, the mere presence of predicted epitopes does not guarantee vaccine suitability. Limited *in vivo* expression during infection may restrict antigen availability and consequently reduce immunogenic efficacy (62). These observations emphasize the importance of preliminary molecular and expression profiling of candidate antigens before short-chain epitope-based screening.

Antibodies against the ferric transporter (FepA; 80–82 kDa) have been previously reported in the sera of some of the *K. pneumoniae* sepsis and urinary tract infected patients (19, 20), Nevertheless, FepA is a metal-regulated protein whose expression depends on iron availability and may not be constitutive, unlike structural outer membrane porins. In the same context, the amount of expressed FepA required for iron intake from the host tissues is not abundant such as that of the structural osmoporins detected by our experiments (64). This likely explains the reasons why our experiments did not recognize an immune response against any of the metal-regulated transporter proteins that were investigated elsewhere by *in silico* methods (65), including transporters expressed exclusively during the scarcity of iron, silver, copper, or phosphate in the *Klebsiella* growth medium. These findings altogether reinforce that epitope prediction alone is insufficient for antigen selection; antigen abundance, exposure, and *in vivo* expression dynamics along different types of infections are critical determinants of immunogenic relevance (62).

#### 4.1.5 Expression of bacterial immunomes vary along time and infection site

The abundant expression of exposed epitopes is counteracted by the initiation of the immune response against them. Our findings demonstrate that bacterial immunome expression varies according to infection stage and anatomical site, reflecting adaptive strategies followed by the pathogen that facilitate colonization at specific times post-infection. Following periurethral introduction, *K. pneumoniae* initially colonizes the urethra and subsequently ascends to the bladder, where it overexpresses type 1 fimbriae (FIM) to adhere to and invade superficial umbrella cells (66, 67). Simultaneously, the UT-model exhibited an approximately fivefold higher IgM titer (**Figure 2A**) compared with the RT-model and the SP-model (**Figures 3A** and **4A**). This heightened response likely reflects the pathogenic requirement for fimbriae protein (FIM) overexpression via the FIM-switch-system to ensure successful urinary tract colonization (13, 32, 56, 66), which, in turn, provokes a strong primary IgM response. Moreover, the IgA titer level reported against FIM was the second highest after the CPS (**Figure 2B**). Additionally, the IgA titers against FIM were the second highest after CPS in the UT-model (**Figure 2B**), reinforcing the major role of secretory IgA in mucosal urinary tract immunity (67–69). Although type 1 fimbriae are mainly expressed in the urinary tract, limited expression in the respiratory tract is necessary for bacterial persistence (70), explaining the remarkable immune response against FIM in the RT-model (**Figures 3A, C**). A notable correlation was observed between FIM and CPS due to their co-action in inhibiting the immune system and their correlated tendency to produce biofilm that protects the pathogen from immunity (56). Moreover, CPS confers the protection from phagocytosis that was triggered by the overexpression of FimH type 1 adhesin (59, 71). This synergistic interaction supports bacterial survival while shaping the magnitude and quality of the host immune response.

#### 4.1.6 Immune response tends to avoid immunity disarmament by the pathogen

The capsule is the most extensively studied and outermost virulence factor for *K. pneumoniae*. CPS enables the bacterium to resist antimicrobial peptides, antibiotics, and phagocytosis as well as allows evasion and persistence in immune cells, particularly in hypervirulent K1/K2 strains (71, 72). In our study, a significant increase in IgM, IgA, and IgG against CPS was observed exclusively in the UT-model (**Figure 2**), closely correlating with responses against FIM. This reflects immune counteraction of CPS overexpression, which facilitates resistance to phagocytosis and synergizes with fimbriae to establish protective biofilms (56, 71). Consistently, CPS induced the highest lymphocyte proliferation within the UT-model (**Figure 5A**).

This means that, at mucosal surfaces, *K. pneumoniae* co-overexpresses fimbrial adhesins and CPS both to promote adhesion despite mucosal washing and innate opsonization and to prevent phagocytic clearance, respectively. Together they contribute to biofilm formation and immune shielding. This coordinated virulence strategy is met by rapid IgM and IgA responses that block adhesins, limit attachment, and disrupt biofilm establishment. In contrast, the pathogen infections at non-mucosal sites such as the peritoneum do not require strong adhesin expression or biofilm formation. Outer membrane proteins (OMPs), particularly OmpA and OmpC, are instead overexpressed to modulate TLR activation and reduce phagocytosis, respectively (59, 75). Accordingly, immune responses in the SP-model were primarily directed against OMPs, along with strong neutralizing responses to LPS. Notably, at day 3 post-infection, the IgM response against OMP in the RT-model (**Figure 3A**) exceeded that of the UT-model, likely reflecting preferential OMP overexpression in the respiratory tract (56), where FIM and CPS play a less dominant role (69, 73, 74). This predicts that the immune system tailors its capabilities to deactivate the virulence factors that counteract the system activity to clear the infection while giving priorities to the most harmful ones which is the OMP, followed by LPS in the case of the RT-model.

Lipopolysaccharide (LPS), a partially hydrophobic and potent TLR4-activating virulence factor, protects *K. pneumoniae* from complement-mediated killing while posing a risk of systemic inflammation. In the SP-model, both Western blot and ELISA demonstrated a remarkable and progressive systemic response against LPS, reaching approximately eightfold (IgM) and 4.4-fold (IgG) increases at 6 weeks post-infection (**Figures 4A, C**). This remarkable increase in antibody response likely aims to mask the O-antigens that protect the bacterium from the complement system (55, 59) and to instantly neutralize the endotoxic effect of LPS as soon as they dissociate from the bacterial cell in order to prevent a possible cytokine storm and septic shock. Overall, these findings indicate that the immune system adapts its responses according to site-specific virulence strategies, prioritizing neutralization of the most functionally dominant virulence factors of the pathogen at each infection niche.

#### 4.1.7 Downregulation of some antigens

Although FIM strongly stimulated antibody production in the UT-model, thus preventing bacterial attachment to the mucosa, its capacity to induce a sustained cellular immune response was limited. This observation appears inconsistent with the reported ability of FIM to bind antigen-presenting cells (APCs) and provisionally activate cellular immunity (59). However, this reduced cellular response likely refers to the downregulation of FIM production at this stage of infection (59, 70). Fimbriae are predominantly expressed during early colonization and are downregulated once stable attachment is achieved, as continued expression may enhance phagocytic recognition and threaten bacterial survival. Although FIM induced the second-highest lymphocyte proliferation in the SP-model (**Figure 5C**), this response declined and was not enhanced by the booster dose, supporting the notion that FIM expression is transient and stage-specific (59, 70), to avoid triggering phagocytosis that threatens the pathogen’s existence (59). In the RT-model, FIM expression was minimal (**Figure 5B**) and insufficient to trigger a meaningful cellular immune response (74). Several studies identified vaccine candidates against *K. pneumoniae* infection of the urinary tract based on *in silico* reverse vaccinology screening (70, 76) or molecular techniques (19). However, these efforts have focused on type 1 fimbriae despite their expression being limited to a specific period of infection.

#### 4.1.8 Energy management by the immune system

The study highlights the immune system’s capability to prioritize its responses toward the most threatening immunomes while minimizing unnecessary energy expenditure. In non- or partially mucosal sites (SP-model and RT-model), the induction of strong mucosal IgA responses appears unnecessary. Accordingly, IgA responses against all tested antigens displayed irregular and modest patterns in the SP-model and the RT-model (**Figures 3B** and **4B**), indicating that IgA is not the principal mediator of protection in these settings. This distinction underscores the compartmentalization of immune strategies: mucosal surfaces such as the urinary (and likely genital) tract predominantly depend on IgA-mediated defense, whereas respiratory (partially mucosal) and systemic sites rely more on alternative humoral and cellular mechanisms (67–69). These findings confirm the body’s tendency to tailor an immune response against the most threatening or overexpressed antigens with minimal waste of energy—for example, in systemic infection, a robust IgM/IgG response against LPS is sufficient to neutralize endotoxin and promote clearance without the need to expend more resources on strong mucosal IgA production or extensive cellular activation.

#### 4.1.9 Red lines for the immune system

The peritoneal cavity contains the omentum, an adipose-rich structure harboring “milky spots,” which are leukocyte aggregates that serve as key initiators of peritoneal immune responses (77). Upon that evidence, the pronounced response to LPS in the SP-model may partly relate to the non-polar nature of the LPS that binds to the non-polar adipose tissue harboring the leukocytes (10). In addition, the cholesterol-rich adipose tissue enhances macrophage-mediated phagocytosis (32) and synthetizes the immune system. The sustained close contact between the non-polar component of the pathogen and the immune cell initiates a relatively slower and delayed (78) yet high immune response due to the TLR4 and complement activation capacities of LPS (59, 71). Unlike proteins, LPS is not overexpressed at any particular site and time. However, given the anatomical proximity of the peritoneal cavity to vital internal organs, there is an urgency for the immune system to neutralize the endotoxic LPS by a strongly persistent antibody response more than any other site (77). Accordingly, the SP-model demonstrated the tendency of the immune system to prioritize its actions against the most endangering antigens, which is the LPS in the case of the SP-model.

Overall, *K. pneumoniae* infections at external barrier sites such as the urinary and respiratory tracts predominantly elicited early protective IgM responses (**Figures 2A** and **3A**), complemented by IgA at fully mucosal sites such as the UT-model (**Figure 2B**) (68). These antibodies primarily target virulence determinants that impair host defenses: O-antigen, which limits complement activity (59); OMPs, which interfere with TLR activation and phagocytosis (59, 75); CPS which prevents engulfment by immune cells (72); and when occasionally expressed, FIM, which mediates adhesion and immune modulation (69, 73, 74). At mucosal barrier sites, neutralizing antibodies directed against these factors are generally sufficient to control infection (67, 74, 77). In contrast, the systemic SP-model showed a remarkable protective increase in both IgM and IgG titers as being the second line non-mucosal barrier (55) closely located in contact with critical organs. In another way, once infection breaches primary barriers, there is greater urgency for sustained IgG-mediated protection. In this setting, the long-acting protective immune response is mainly due to the cellular response expressed against the OMP in particular. Collectively, these findings confirm that the immune system tailors its responses according to many criteria such as antigen abundance, degree of epitopes’ exposure to the immune system, site of the infection, and the relative threat posed by that specific virulence factor to limit the danger of pathogen propagation or harm for the immune system.

### 4.2 Fingerprinting the infection and settlement of infection stage

Our findings provide potential evidence supporting the use of distinct immune response profiles against *Klebsiella pneumoniae* infection to determine both the source and the stage of infection. In a broader context, this approach may serve as a forensic or juridical tool to determine whether a patient acquired *K. pneumoniae* infection during hospitalization at a specific healthcare facility and to infer the route by which the pathogen entered the body. Based on our data, quantification of IgM antibodies directed against specific bacterial antigens may help predict both the route and the timing of infection acquisition. The principal advantage of using IgM as a diagnostic criterion for infection staging and medico-legal evaluation lies in its ability to reflect active infection only, regardless of previous exposure to the same pathogen, as IgM gradually declines after the first infection as IgG becomes immunodominant (55). A comparative analysis of IgM response profiles (**Figures 2A**, **3A**, and **4A**) may provide an immunological “fingerprint” of the infection route. A urinary tract infection may be indicated by a detectable IgM response against *Klebsiella* fimbrial (FIM) antigens. In contrast, intratracheal infection may be identified by elevated IgM responses directed against outer membrane proteins (OMPs). Septicemia or bloodstream infection due to *K. pneumoniae* may be recognized by measuring IgM titers against the lipopolysaccharide (LPS) O-antigen. Importantly, bloodstream infection may arise either as a primary event (e.g., direct contamination via a central venous catheter) or secondarily from dissemination from another anatomical site (10). In such cases, correlating IgM responses against the three proposed antigenic targets may provide a more precise indication of the infection source. The progressive fold-increase in IgM titers against these antigens may help estimate the timing of primary infection acquisition. Serial measurement of IgM titers at two to three time points, separated by 3-day intervals, may be sufficient to determine both infection stage and probable source. Ultimately, this approach could facilitate the development of a predictive mathematical model to estimate the infection stage based on dynamic IgM kinetics.

### 4.3 Further applications for the study outcomes

The development of a pathogen-specific diagnostic tool is typically based on detecting the earliest antibody isotype elicited during infection, provided that it is produced at sufficiently measurable levels. Concurrently, the antigen selected as the diagnostic core must exhibit high pathogen specificity and a consistent expression across different anatomical niches (15). Our experimental findings confirm that IgM is the most appropriate immunoglobulin for this purpose. IgM was robustly induced against multiple antigens across all investigated models of *Klebsiella pneumoniae* infection, demonstrating a rapid twofold increase as early as day 3 post-infection (**Figures 2A**, **3A**, and **4A**). While protein antigens may undergo downregulation for variable reasons and capsular polysaccharide (CPS) serotypes exhibit high variability, lipopolysaccharide (LPS) emerges as a comparatively stable diagnostic target. Among the evaluated antigens, LPS elicited the earliest and highest IgM responses across the different infection models. Although the conserved core moiety of LPS has limited specificity as being partially shared among several members of Enterobacteriaceae (79), the O-antigen moieties of *K. pneumoniae* LPS provides a critical discriminatory advantage, effectively serving as an “Achilles’ heel” for selective pathogen detection. The conclusive results for using O-antigen consolidate previous findings in the field (20).

An effective vaccine must meet fundamental criteria, including safety, ease of production, cost-effectiveness, broad-spectrum coverage, and the ability to induce long-term humoral and cellular immune protection (15, 16). Moreover, the target antigens should be sufficiently abundant and surface-exposed to trigger a quick and appropriate immune response (62). In *K. pneumoniae*, fimbriae (FIM) expression can be downregulated by antibiotic pressure or masked by certain capsular polysaccharide (CPS) serotypes (59, 80) or restricted to specific infection sites. Although FIM demonstrated a favorable safety profile, its limited capability to induce sustained lymphocyte proliferation across all infection models (**Figure 5**) suggests insufficient long-term protective immunity. Capsular polysaccharides induced stronger lymphocyte proliferation, highlighting CPS as a potential vaccine candidate. Nevertheless, the presence of more than 80 CPS serotypes presents a substantial obstacle to practical vaccine development and broad-spectrum applicability (10, 13). Additionally, CPS-mediated impairment of phagocytosis further limits its potential use for vaccine production (72).

Lipopolysaccharide (LPS) therefore presents a compelling alternative vaccine component, as antibodies targeting LPS have been shown to mediate effective opsonization and neutralization for antigen endotoxicity (10, 13, 20). Moreover, the relatively limited diversity of *K. pneumoniae* O-antigens enhances the feasibility of developing a broad-spectrum vaccine. Nevertheless, although detoxified LPS has been proposed for vaccine use (17), its capacity to induce long-lasting T cell-dependent cellular immunity remains limited (59).

Outer membrane proteins (OMPs) elicited the most robust lymphocyte proliferation across all infection routes, accompanied by early protective antibody production. However, key OMPs such as OmpK17/X, OmpK35/F, and the non-metal-regulated OmpK36/C are poorly expressed in bacteriocin-producing, extended-spectrum β-lactamase (ESBL) and carbapenem-resistant *K. pneumoniae* strains (81, 82), limiting their applicability. Furthermore, OmpA, a major virulence-associated OMP, has been shown to suppress cytokine production and attenuate cellular immune responses (59), thus reducing its suitability as a vaccine antigen. To harness the immunological strengths of these antigen classes while mitigating their individual limitations, we propose a conjugate vaccine strategy combining detoxified LPS with selected OMPs. Such an approach has the potential to provide broad-spectrum, long-term protection and represents a rational vaccine design strategy against *K. pneumoniae*. Despite the pioneering impact of this model-leading study in demonstrating the hypothesis that antigen expression varies according to the site and stage of infection, the screening of a large number of sampling points led to certain limitations in some research aspects that should be considered in future studies. The immunoblotting results showed a degree of overlap, which may have reduced the clarity of interpretation and the overall consistency of the findings. Therefore, future investigations should consider the use of two-dimensional electrophoresis or HPLC-based separation of the target antigens already proposed in our study in order to evaluate the immune potency of the individual components of each antigen through quantitative ELISA assays. Moreover, developing the analytical capacity will also have to include detailed cytokine profiling, analysis of specific antibody subclasses, and investigation of innate immune responses against those individual antigenic components. This addition may provide more comprehensive insights beyond the current screening of overall immune activity against whole antigens alone. Our study leads the path for future studies developed for epitope mapping of pathogens for a more precisely efficient *in silico* screening of epitope in the proposed antigens, for studies of the mechanisms of virulence factor expression at different anatomical sites, and for the development of diagnostic tools and vaccines.

## 5 Conclusion

*Klebsiella pneumoniae* exhibits dynamic, site- and stage-specific interactions with the host immune system, actively modulating antigen expression. Overexpression, downregulation, or even hiding of antigenic virulence factors is controlled by pathogens to secure successful battle outcomes against the immune system and to induce infection. Subsequently, the actions of the pathogen are counteracted by tailored immune responses to control the invasion in a dilemma resembling the “chess game”. This study demonstrates that understanding the temporal and spatial signatures of host immunity is critical for rational vaccine and diagnostic design. Our findings identify conjugate lipopolysaccharides (LPS) and outer membrane proteins (OMPs) as promising vaccine antigens, while O-antigens provide highly suitable targets for diagnostics. We further show that immune profiling can discriminate both the stage and route of infection, highlighting the value of combining polysaccharide and protein markers.

While computational epitope prediction remains a powerful tool for identifying short, immunogenic, safe, and broadly conserved peptide sequences within validated protein antigens, it cannot capture the dynamic context of infection or polysaccharide epitopes in complex pathogens like bacteria. Therefore, integrating *in vivo* molecular mapping with *in silico* epitope design provides a necessary framework for the development of broad-spectrum protective vaccines and targeted diagnostics. This innovative approach offers a strategic pathway to overcome the challenges of complex bacterial pathogens and advance precision immunological interventions against *K. pneumoniae.*

## Data Availability

The datasets presented in this study can be found in online repositories. The names of the repository/repositories and accession number(s) can be found in the article/**Supplementary Material**.
