# Supplementary material for: Variable progressive behavior of Klebsiella pneumoniae at different sites of infection
Source: Front Immunol. 2026 Apr 13;17:1775450. doi: 10.3389/fimmu.2026.1775450 (PMC13111035; doi:10.3389/fimmu.2026.1775450)
Supplement: Supplementary file 1 [file DataSheet1.pdf]

Supplementary File (3)
Statistical Data

	Fold increase									
	W0	W½	W1	W2	W2½	W3	W4	W5	W6	W7
Omp	1	1.970085	5.233573	2.979621	2.728543	3.152789	2.796734	2.755715	3.448334	2.311561
FIM	1	4.150315	10.81817	4.324243	5.907752	6.011503	4.868426	4.90134	4.890883	2.913284
CPS	1	2.959805	8.880595	4.172483	4.794039	5.856378	4.803484	4.600357	4.049955	2.726295
LPS	1	2.247336	5.919409	3.106106	3.240642	3.490073	2.940801	3.25439	3.195633	2.485638
FIWC	1	1.374621	4.048151	2.227545	1.926059	1.95682	1.781445	1.914708	2.076575	1.283018

	SE									
	W0	W½	W1	W2	W2½	W3	W4	W5	W6	W7
Omp	0.005406	0.01696	0.072716	0.022955	0.008638	0.002862	0.031669	0.025786	0.006257	0.016584
FIM	0.011454	0.03573	0.304155	0.02877	0.05939	0.006481	0.05652	0.029024	0.107081	0.048262
CPS	0.003677	0.055081	0.067196	0.025097	0.087024	0.063518	0.084275	0.04744	0.023673	0.011852
LPS	0.011056	0.034583	0.039899	0.068051	0.067758	0.056728	0.035074	0.023816	0.008948	0.032682
FIWC	0.402041	0.023598	0.026093	0.014658	0.028659	0.009896	0.03076	0.035373	0.038635	0.01775

	p-value (Every sample, by paired t-test)									
	W0	W½	W1	W2	W2½	W3	W4	W5	W6	W7
Omp	-	0.000335	0.000605	0.002615	0.021746	0.000961	0.013254	0.111013	0.001419	0.00027
FIM	-	0.00852	0.003013	0.003625	0.002075	0.299647	0.004473	0.597079	0.952175	0.003966
CPS	-	0.001342	0.000151	0.000494	0.04494	0.015666	0.004791	0.070838	0.003429	0.000761
LPS	-	0.000633	0.000617	0.001913	0.348019	0.243012	0.037245	0.047427	0.171409	0.003897
FIWC	-	0.546095	0.004782	0.000265	0.003653	0.329083	0.020632	0.06884	0.065868	0.006468

	p-value (every two samples, by paired t-test)									
	W0	W½	W1	W2	W2½	W3	W4	W5	W6	W7
Omp	-	-	0.000379	-	0.001099	-	0.234852	-	0.002341	-
FIM	-	-	0.001438	-	0.004393	-	0.004322	-	0.920895	-
CPS	-	-	0.000114	-	0.000216	-	0.965851	-	0.009629	-
LPS	-	-	0.000153	-	0.000604	-	0.030746	-	0.027686	-
FIWC	-	-	0.028339	-	0.000418	-	0.005231	-	0.052348	-

Urinary tract infection model (IgM): Data for Figure (2A), for the values of the fold increase, Standard Error (SE), and p-values along 7 weeks (W0 till W7) for the Outer Membrane Proteins (Omp), Fimbriae proteins (Fim), Capsular Polysaccharides (CPS), Lipopolysaccharides (LPS), and Formalin Inactivated Whole bacterial Cells (FIWC).

	Fold increase									
	W0	W½	W1	W2	W2½	W3	W4	W5	W6	W7
Omp	1	4.611563	7.798451	6.762944	6.472651	5.622717	6.878866	6.997224	6.907603	6.913448
FIM	1	6.368467	10.51994	8.380305	8.785902	7.474945	9.446425	9.187462	9.644851	9.085222
CPS	1	7.629077	14.36861	11.98703	11.68311	9.67031	12.31049	11.87417	12.07767	11.86173
LPS	1	4.970229	8.891547	6.890484	6.458267	5.038809	6.18235	6.217438	7.192451	6.922913
FIWC	1	3.077008	5.293239	3.753076	3.395548	2.825107	3.160514	3.972625	4.014655	4.01742

	SE									
	W0	W½	W1	W2	W2½	W3	W4	W5	W6	W7
Omp	0.019325	0.122726	0.123909	0.009352	0.118072	0.044518	0.124846	0.049309	0.124126	0.05232
FIM	0.007255	0.169482	0.126792	0.088741	0.155385	0.178536	0.073198	0.151062	0.225407	0.153219
CPS	0.014893	0.031602	0.051593	0.197937	0.206589	0.109252	0.212521	0.050207	0.092576	0.169778
LPS	0.030907	0.052749	0.112145	0.058558	0.0623	0.026744	0.1747	0.140942	0.097454	0.068974
FIWC	0.01118	0.045597	0.03135	0.032009	0.029498	0.036231	0.057984	0.097464	0.060719	0.034298

	p-value (Every sample, by paired t-test)									
	W0	W½	W1	W2	W2½	W3	W4	W5	W6	W7
Omp	-	0.002119	0.002195	0.023779	0.180173	0.03312	0.014958	0.338939	0.663724	0.95932
FIM	-	0.001439	0.006865	0.000743	0.307759	0.060273	0.00618	0.283032	0.042153	0.209323
CPS	-	0.003654	0.000122	0.009363	0.595399	0.032822	0.003866	0.268131	0.145159	0.355714
LPS	-	0.000391	0.00157	0.00409	0.023724	0.000971	0.037841	0.935287	0.056874	0.016605
FIWC	-	0.000639	0.001286	0.004782	0.007465	0.010388	0.012576	0.048906	0.835456	0.979974

	p-value (every two samples, by paired t-test)									
	W0	W½	W1	W2	W2½	W3	W4	W5	W6	W7
Omp	-	-	0.000663	-	0.019593	-	0.006496	-	0.907814	-
FIM	-	-	0.000294	-	0.036891	-	0.072108	-	0.54541	-
CPS	-	-	0.000254	-	0.00886	-	0.346073	-	0.592372	-
LPS	-	-	0.000159	-	0.000721	-	0.376055	-	0.026928	-
FIWC	-	-	0.000147	-	0.000506	-	0.067989	-	0.001233	-

Urinary tract infection model (IgA): Data for Figure (2B), for the values of the fold increase, Standard Error (SE), and p-values along 7 weeks (W0 till W7) for the Outer Membrane Proteins (Omp), Fimbriae proteins (Fim), Capsular Polysaccharides (CPS), Lipopolysaccharides (LPS), and Formalin Inactivated Whole bacterial Cells (FIWC).

	Fold increase									
	W0	W½	W1	W2	W2½	W3	W4	W5	W6	W7
Omp	1	1.306799	1.756893	1.589258	1.498814	1.374069	1.390616	1.518162	1.878099	1.623415
FIM	1	1.428373	1.88162	1.590947	1.6239	1.445275	1.45757	1.637805	1.807219	1.505547
CPS	1	1.338571	1.830928	1.57599	1.520959	1.439873	1.442838	1.632817	1.733624	1.402766
LPS	1	1.339154	1.819241	1.626238	1.551096	1.434282	1.431314	1.629947	1.682747	1.460332
FIWC	1	1.303502	1.658684	1.482336	1.434046	1.277094	1.343795	1.600182	2.069517	1.746115

	SE									
	W0	W½	W1	W2	W2½	W3	W4	W5	W6	W7
Omp	0.003154	0.02049	0.009203	0.007561	0.011106	0.003984	0.005193	0.017611	0.01709	0.012731
FIM	0.006052	0.022396	0.021558	0.007962	0.008844	0.033406	0.009438	0.001654	0.014895	0.012338
CPS	0.002384	0.025696	0.009388	0.021169	0.020473	0.018425	0.021717	0.00419	0.01451	0.005558
LPS	0.009775	0.033994	0.009887	0.004968	0.00593	0.007206	0.009665	0.011888	0.01364	0.010531
FIWC	0.00787	0.011305	0.010488	0.014113	0.01041	0.00329	0.006376	0.004558	0.025327	0.007325

	p-value (Every sample, by paired t-test)									
	W0	W½	W1	W2	W2½	W3	W4	W5	W6	W7
Omp	-	0.005604	0.001531	0.000996	0.005681	0.01108	0.237716	0.029591	0.00947	0.006532
FIM	-	0.002939	0.012476	0.004881	0.150786	0.074632	0.826266	0.003669	0.01005	0.009602
CPS	-	0.007985	0.002489	0.003188	0.375919	0.13606	0.814888	0.013355	0.01563	0.001544
LPS	-	0.009795	0.003951	0.001527	0.028681	0.01552	0.894494	0.016391	0.231612	0.008415
FIWC	-	0.000226	0.001241	0.000722	0.231955	0.007713	0.028891	0.000583	0.00363	0.00575

	p-value (Every two samples, by paired t-test)									
	W0	W½	W1	W2	W2½	W3	W4	W5	W6	W7
Omp	-	-	0.000254	-	0.005912	-	0.009298	-	0.00304	-
FIM	-	-	0.001003	-	0.006259	-	0.002873	-	0.005543	-
CPS	-	-	0.000239	-	0.012639	-	0.10545	-	0.001427	-
LPS	-	-	0.000214	-	0.005171	-	0.011538	-	0.001667	-
FIWC	-	-	0.00035	-	0.010978	-	0.014047	-	0.002231	-

Urinary tract infection model (IgG): Data for Figure (2C), for the values of the fold increase, Standard Error (SE), and p-values along 7 weeks (W0 till W7) for the Outer Membrane Proteins (Omp), Fimbriae proteins (Fim), Capsular Polysaccharides (CPS), Lipopolysaccharides (LPS), and Formalin Inactivated Whole bacterial Cells (FIWC).

	Fold increase									
	W0	W½	W1	W2	W2½	W3	W4	W5	W6	W7
Omp	1	3.856641	3.885696	3.952427	3.048531	2.393678	2.354406	2.180396	1.534483	1.736271
FIM	1	1.75977	1.745977	1.744253	1.711207	1.456609	1.506034	1.554023	0.90977	1.195402
CPS	1	1.841987	1.792737	1.889848	2.369854	1.829501	1.789963	1.404294	1.253078	1.438283
LPS	1	2.078165	2.170543	2.25646	1.459302	1.303295	1.514535	1.869186	1.464147	1.827519
FIWC	1	1.671969	1.638244	1.693605	1.019408	1.085269	1.156538	1.324849	1.053134	1.225581

	SE									
	W0	W½	W1	W2	W2½	W3	W4	W5	W6	W7
Omp	0.030009	0.054489	0.026703	0.069987	0.150746	0.029277	0.063772	0.037071	0.043124	0.02621
FIM	0.025379	0.019852	0.012342	0.029187	0.033085	0.017514	0.042188	0.00765	0.030897	0.00807
CPS	0.037387	0.029209	0.031241	0.02266	0.07879	0.032654	0.013255	0.02404	0.023733	0.037142
LPS	0.0216	0.003831	0.060443	0.021471	0.002417	0.030234	0.027875	0.013997	0.041588	0.04623
FIWC	0.038854	0.011839	0.016778	0.020255	0.03476	0.012599	0.015783	0.040653	0.036655	0.038489

	p-value (Every sample, by paired t-test)									
	W0	W½	W1	W2	W2½	W3	W4	W5	W6	W7
Omp	-	0.000421	0.765145	0.62914	0.068156	0.08753	0.745188	0.293714	0.022034	0.139617
FIM	-	0.003533	0.758425	0.973686	0.648604	0.010181	0.323913	0.513136	0.002208	0.017278
CPS	-	0.00183	0.432581	0.228676	0.052293	0.017657	0.263581	0.012341	0.053118	0.01671
LPS	-	0.000631	0.357982	0.430445	0.000917	0.046636	0.058811	0.010336	0.011215	0.037162
FIWC	-	0.003032	0.08505	0.324698	0.006944	0.278886	0.108793	0.133679	0.096407	0.196574

	p-value (every two samples, by paired t-test)									
	W0	W½	W1	W2	W2½	W3	W4	W5	W6	W7
Omp	-	-	0.000112	-	0.035225	-	0.02304	-	0.00171	-
FIM	-	-	0.001241	-	0.318923	-	0.003518	-	0.01894	-
CPS	-	-	0.001116	-	0.040444	-	0.019222	-	0.006385	-
LPS	-	-	0.00589	-	0.011433	-	0.269211	-	0.199589	-
FIWC	-	-	0.004614	-	0.007172	-	0.028073	-	0.061489	-

Respiratory tract infection model (IgM): Data for Figure (3A), for the values of the fold increase, Standard Error (SE), and p-values along 7 weeks (W0 till W7) for the Outer Membrane Proteins (Omp), Fimbriae proteins (Fim), Capsular Polysaccharides (CPS), Lipopolysaccharides (LPS), and Formalin Inactivated Whole bacterial Cells (FIWC).

	Fold increase									
	W0	W½	W1	W2	W2½	W3	W4	W5	W6	W7
Omp	1	1.492045	1.389773	1.363149	1.186688	1.423864	1.224513	1.334903	1.266234	1.12013
FIM	1	1.501062	1.383146	1.26082	1.231259	1.379553	1.253634	1.33611	1.472807	1.359464
CPS	1	1.374559	1.373686	1.350285	1.319549	1.581503	1.276414	1.369495	1.580455	1.482833
LPS	1	1.369655	1.322759	1.316034	1.113103	1.533621	1.376034	1.55569	1.494828	1.422759
FIWC	1	1.339711	1.42322	1.380412	1.117237	1.485649	1.346198	1.621534	1.457435	1.400843

	SE									
	W0	W½	W1	W2	W2½	W3	W4	W5	W6	W7
Omp	0.018977	0.041358	0.023164	0.024649	0.044969	0.014179	0.048569	0.003862	0	0
FIM	0.020376	0.041608	0.030856	0.026733	0.042023	0.012972	0.018028	0.018723	0.044082	0.017125
CPS	0.003332	0.006201	0.01034	0.015562	0.028844	0.089247	0.028727	0.024562	0.03753	0.022147
LPS	0.008169	0.031492	0.012195	0.0111	0.011538	0.013523	0.013686	0.010729	0.020141	0.008863
FIWC	0.012044	0.023066	0.009997	0.020642	0.015715	0.005164	0.007607	0.035029	0.001213	0.019472

	p-value (Every sample, by paired t-test)									
	W0	W½	W1	W2	W2½	W3	W4	W5	W6	W7
Omp	-	0.009277	0.251291	0.561726	0.171984	0.032731	0.042589	0.182757	0.004711	0.1923
FIM	-	0.010828	0.274604	0.144175	0.759076	0.074666	0.009688	0.183684	0.207884	0.167467
CPS	-	0.000804	0.957708	0.178733	0.323567	0.129781	0.058606	0.025734	0.029116	0.053633
LPS	-	0.006147	0.198216	0.785633	0.007277	0.000138	0.001263	0.016641	0.126652	0.145082
FIWC	-	0.001684	0.102181	0.347031	0.017379	0.002018	0.008547	0.01966	0.06357	0.145385

	p-value (every two samples, by paired t-test)									
	W0	W½	W1	W2	W2½	W3	W4	W5	W6	W7
Omp	-	-	0.017042	-	0.072971	-	0.539098	-	0.555698	-
FIM	-	-	0.024659	-	0.128018	-	0.544009	-	0.056067	-
CPS	-	-	0.001854	-	0.254178	-	0.490373	-	0.005186	-
LPS	-	-	0.000234	-	0.000649	-	0.00137	-	0.01265	-
FIWC	-	-	0.002077	-	0.001303	-	0.015036	-	0.009281	-

Respiratory tract infection model (IgA): Data for Figure (3B), for the values of the fold increase, Standard Error (SE), and p-values along 7 weeks (W0 till W7) for the Outer Membrane Proteins (Omp), Fimbriae proteins (Fim), Capsular Polysaccharides (CPS), Lipopolysaccharides (LPS), and Formalin Inactivated Whole bacterial Cells (FIWC).

	Fold increase									
	W0	W½	W1	W2	W2½	W3	W4	W5	W6	W7
Omp	1	1.671146	1.611172	1.574877	1.46279	1.495703	1.305632	1.42942	1.411501	1.491589
FIM	1	2.411382	2.14068	1.693073	1.614976	1.743467	1.505613	1.664446	1.794652	1.735024
CPS	1	2.023601	1.857471	1.770464	1.689846	1.836535	1.532553	1.772911	1.840477	1.745721
LPS	1	1.942023	1.744807	1.551468	1.372135	1.430161	1.266586	1.37251	1.637507	1.61187
FIWC	1	1.481518	1.329696	1.23904	1.236064	1.231369	1.297891	1.485618	1.072142	1.071097

	SE									
	W0	W½	W1	W2	W2½	W3	W4	W5	W6	W7
Omp	0.007125	0.013448	0.004931	0.023848	0.055003	0.009827	0.017541	0.006893	0.028409	0.007351
FIM	0.005939	0.019405	0.148804	0.012834	0.017351	0.001413	0.024975	0.024007	0.006437	0.02295
CPS	0.002671	0.021925	0.022798	0.027704	0.006755	0.001709	0.012317	0.023436	0.040593	0.013293
LPS	0.003283	0.011314	0.014203	0.012596	0.010089	0.000613	0.095258	0.012326	0.01737	0.015194
FIWC	0.008248	0.008752	0.011362	0.007262	0.016872	0.053549	0.003556	0.007483	0.000378	0.009267

	p-value (Every sample, by paired t-test)									
	W0	W½	W1	W2	W2½	W3	W4	W5	W6	W7
Omp	-	0.000278	0.116665	0.344239	0.356009	0.709678	0.007214	0.053655	0.67909	0.160476
FIM	-	0.000405	0.26747	0.139405	0.030721	0.027013	0.017934	0.033774	0.07088	0.214702
CPS	-	0.00068	0.058426	0.059765	0.127272	0.004714	0.002139	0.009332	0.403574	0.252311
LPS	-	0.000199	0.011714	0.024301	0.022987	0.046683	0.29492	0.496146	0.013809	0.092805
FIWC	-	0.001696	0.020695	0.028876	0.906143	0.955386	0.420233	0.004834	0.000528	0.935117

	p-value (every two samples, by paired t-test)									
	W0	W½	W1	W2	W2½	W3	W4	W5	W6	W7
Omp	-	-	0.00051	-	0.149946	-	0.218611	-	0.093742	-
FIM	-	-	0.023351	-	0.119893	-	0.102986	-	0.016337	-
CPS	-	-	0.000835	-	0.033898	-	0.01177	-	0.01837	-
LPS	-	-	0.00081	-	0.001368	-	0.49463	-	0.062016	-
FIWC	-	-	0.002574	-	0.113275	-	0.129809	-	0.000299	-

Respiratory tract infection model (IgG): Data for Figure (3C), for the values of the fold increase, Standard Error (SE), and p-values along 7 weeks (W0 till W7) for the Outer Membrane Proteins (Omp), Fimbriae proteins (Fim), Capsular Polysaccharides (CPS), Lipopolysaccharides (LPS), and Formalin Inactivated Whole bacterial Cells (FIWC).

	Fold increase									
	W0	W½	W1	W2	W2½	W3	W4	W5	W6	W7
Omp	1	1.191479	2.333414	2.739877	3.144931	2.541574	2.424436	2.604304	2.583693	2.540934
FIM	1	1.701245	2.822125	2.141591	2.382328	1.683331	2.41651	2.52911	1.540936	1.15488
CPS	1	1.288454	1.430468	1.854666	2.225194	1.308373	1.999262	1.763003	1.496311	1.414976
LPS	1	1.262206	2.727835	2.983525	6.010666	6.005015	7.122248	8.030054	9.350886	6.316682
FIWC	1	1.296857	1.510186	1.808692	1.949166	0.973419	1.650757	1.487582	1.603803	1.40163

	SE									
	W0	W½	W1	W2	W2½	W3	W4	W5	W6	W7
Omp	0.001199	0.016311	0.050038	0.023821	0.15623	0.008519	0.019232	0.03271	0.008571	0.005017
FIM	0.005435	0.023289	0.470345	0.009896	0.291788	0.016103	0.024861	0.004104	0.004477	0.02124
CPS	0.006348	0.003819	0.037875	0.001137	0.05266	0.007917	0.013766	0.001528	0.013728	0.044238
LPS	0.0011	0.005789	0.006363	0.023278	0.114444	0.010058	0.032114	0.054445	0.083134	0.032214
FIWC	0.008832	0.02032	0.015404	0.010842	0.03055	0.011649	0.011498	0.004926	0.016703	0.058034

	p-value (Every sample, by paired t-test)									
	W0	W½	W1	W2	W2½	W3	W4	W5	W6	W7
Omp	-	0.010198	0.003612	0.045729	0.144711	0.088089	0.015707	0.103991	0.704684	0.099217
FIM	-	0.001296	0.202021	0.352058	0.571395	0.197274	0.004601	0.085155	0.009753	0.003014
CPS	-	0.000116	0.0906	0.011489	0.028663	0.00498	0.000624	0.005744	0.004629	0.161595
LPS	-	0.000918	0.005372	0.011649	0.002922	0.969039	0.001303	0.012919	0.010539	0.00043
FIWC	-	0.005072	0.019104	0.008477	0.103525	0.002788	0.006754	0.008775	0.024134	0.155059

	p-value (every two samples, by paired t-test)									
	W0	W½	W1	W2	W2½	W3	W4	W5	W6	W7
Omp	-	-	0.002025	-	0.070533	-	0.058978	-	0.010424	-
FIM	-	-	0.086996	-	0.653061	-	0.931107	-	0.000858	-
CPS	-	-	0.011066	-	0.00058	-	0.042588	-	0.002602	-
LPS	-	-	2.59E-05	-	0.001743	-	0.012736	-	0.002542	-
FIWC	-	-	0.000422	-	0.002717	-	0.028458	-	0.296927	-

Sepsis infection model (IgM): Data for Figure (4A), for the values of the fold increase, Standard Error (SE), and p-values along 7 weeks (W0 till W7) for the Outer Membrane Proteins (Omp), Fimbriae proteins (Fim), Capsular Polysaccharides (CPS), Lipopolysaccharides (LPS), and Formalin Inactivated Whole bacterial Cells (FIWC).

	Fold increase									
	W0	W½	W1	W2	W2½	W3	W4	W5	W6	W7
Omp	1	1.99380029	1.522622	1.83419074	1.485292178	1.463922965	1.34626	1.323572088	1.346919931	1.274238
FIM	1	1.947558304	1.392217	1.393377142	1.399433063	1.415796933	1.264141	1.253962118	1.288364901	1.118155
CPS	1	1.655923567	1.366369	1.340382166	2.112866242	1.419146497	1.322293	1.39133758	1.303987261	1.104204
LPS	1	1.554435227	1.20485	1.332354818	1.561582642	1.525462668	1.265858	1.479259732	1.52137843	1.313593
FIWC	1	1.62992126	1.270892	1.308864618	1.568834138	1.511938024	1.303277	1.512319025	1.233299467	0.991999

	SE									
	W0	W½	W1	W2	W2½	W3	W4	W5	W6	W7
Omp	0.006065	0.024216899	0.036705	0.02845623	0.063147318	0.014666605	0.112435	0.009365209	0.03210905	0.012946
FIM	0.008123	0.023655239	0.048534	0.005515997	0.056818435	0.019620958	0.103608	0.016973919	0.005613457	0.053781
CPS	0.011008	0.005945102	0.019735	0.003609092	0.211251318	0.012757138	0.113769	0.022184876	0.027593327	0.033604
LPS	0.011779	0.035801898	0.01046	0.007152731	0.024154808	0.006113047	0.052972	0.010174367	0.014534787	0.024113
FIWC	0.013039	0.012390175	0.014545	0.020333794	0.009649234	0.016669801	0.019093	0.014149371	0.048566265	0.015343

	p-value (Every sample, by paired t-test)									
	W0	W½	W1	W2	W2½	W3	W4	W5	W6	W7
Omp	-	0.000687138	0.004147	0.04949829	0.062854852	0.799494201	0.473503	0.885374897	0.608072183	0.202677
FIM	-	0.001174729	0.02439	0.987612574	0.93222295	0.859065468	0.396706	0.948248522	0.252856694	0.1024
CPS	-	0.000843336	0.004201	0.323709425	0.093587928	0.104324595	0.561345	0.7017757	0.285719971	0.092897
LPS	-	0.00286659	0.025195	0.024352186	0.019525613	0.294110678	0.069657	0.095800281	0.213897915	0.008474
FIWC	-	0.005761	0.001267	0.432418858	0.003518399	0.186167178	0.040943	0.035132526	0.058389705	0.048052

	p-value (every two samples, by paired t-test)									
	W0	W½	W1	W2	W2½	W3	W4	W5	W6	W7
Omp	-	-	0.007798	-	0.461976214	-	0.149037	-	0.997362254	-
FIM	-	-	0.020788	-	0.960317087	-	0.157987	-	0.860106036	-
CPS	-	-	0.010285	-	0.086161953	-	0.133193	-	0.889412918	-
LPS	-	-	0.017217	-	0.010882806	-	0.074114	-	0.085507766	-
FIWC	-	-	0.003902	-	0.006158262	-	0.004109	-	0.251498933	-

Sepsis infection model (IgA): Data for Figure (4B), for the values of the fold increase, Standard Error (SE), and p-values along 7 weeks (W0 till W7) for the Outer Membrane Proteins (Omp), Fimbriae proteins (Fim), Capsular Polysaccharides (CPS), Lipopolysaccharides (LPS), and Formalin Inactivated Whole bacterial Cells (FIWC).

	Fold increase									
	W0	W½	W1	W2	W2½	W3	W4	W5	W6	W7
Omp	1	1.558244	1.818042	2.003503	2.076527	1.98106	2.164331	2.321546	2.575881	2.435297
FIM	1	1.642964	1.80561	1.749509	1.84832	1.498673	1.777444	2.040286	1.875113	1.671823
CPS	1	1.410407	1.548097	1.520027	1.36059	1.218795	1.457339	1.744147	1.741263	1.523688
LPS	1	1.646819	2.166263	2.538569	3.267882	3.521994	3.528242	3.857848	4.338136	3.263165
FIWC	1	1.425047	1.421718	1.405327	1.306727	1.33763	1.442889	1.924193	1.438962	1.328496

	SE									
	W0	W½	W1	W2	W2½	W3	W4	W5	W6	W7
Omp	0.007062	0.014325	0.023891	0.024314	0.0809	0.021322	0.015531	0.020969	0.003228	0.018861
FIM	0.00066	0.015104	0.078908	0.023844	0.199487	0.026678	0.021341	0.017647	0.058739	0.015599
CPS	0.018088	0.005592	0.012437	0.010434	0.039713	0.026705	0.003232	0.011974	0.028721	0.020048
LPS	0.003287	0.001986	0.019399	0.019091	0.04073	0.03243	0.012828	0.037948	0.073776	0.015644
FIWC	0.012714	0.007911	0.02527	0.008998	0.010982	0.007991	0.012457	0.01639	0.04151	0.030071

	p-value (Every sample, by paired t-test)									
	W0	W½	W1	W2	W2½	W3	W4	W5	W6	W7
Omp	-	0.000895	0.030255	0.087668	0.57297	0.517576	0.053719	0.070979	0.008931	0.027232
FIM	-	0.000778	0.199969	0.585077	0.735461	0.33215	0.026467	0.028611	0.16589	0.102959
CPS	-	0.001544	0.011017	0.027434	0.048349	0.114696	0.016977	0.001816	0.945086	0.004852
LPS	-	0.00543	0.002228	0.015552	0.002509	0.037601	0.892445	0.009076	0.04212	0.009681
FIWC	-	0.003452	0.93283	0.506141	0.055503	0.29221	0.020177	0.001816	0.006314	0.312958

	p-value (every two samples, by paired t-test)									
	W0	W½	W1	W2	W2½	W3	W4	W5	W6	W7
Omp	-	-	0.001409	-	0.107885	-	0.387889	-	0.002377	-
FIM	-	-	0.014116	-	0.911496	-	0.788625	-	0.382493	-
CPS	-	-	0.002611	-	0.035759	-	0.203827	-	0.012651	-
LPS	-	-	0.000492	-	0.004111	-	0.017535	-	0.013713	-
FIWC	-	-	0.00384	-	0.122303	-	0.039081	-	0.952564	-

Sepsis infection model (IgG): Data for Figure (4C), for the values of the fold increase, Standard Error (SE), and p-values along 7 weeks (W0 till W7) for the Outer Membrane Proteins (Omp), Fimbriae proteins (Fim), Capsular Polysaccharides (CPS), Lipopolysaccharides (LPS), and Formalin Inactivated Whole bacterial Cells (FIWC).

Stimulation Index									
UTI	W0	W1	W2	W2½	W3	W4	W5	W6	W7
OMP	0	6.211499	7.015743	5.065024	9.514031	7.700205	6.570842	3.755989	6.194387
FIM	0	0.011111	0.127778	1.116667	0	0	0	0	0
LPS	0	1.569534	2.219585	0.527953	1.800769	0.237374	0	2.054514	1.381954
CPS	0	2.727273	0.782828	1.313131	6.515152	2.676768	0	0.10101	2.5
FIWC	0	1.694444	1.197222	0.296296	2.001543	1.018519	0	2.393519	2.25

	SE								
	W0	W1	W2	W2½	W3	W4	W5	W6	W7
Omp	0.028251	0.086668	0.391258	0.070598	0.014756	0.033311	0.056656	0.014846	0.00869
FIM	0.040338	0.081466	0.01714	0.085054	0.027891	0.043061	0.007108	0.007755	0.036789
CPS	0.006614	0.065602	0.001969	0.09121	0.013713	0.023843	0.002647	0.023778	0.076623
LPS	0.010026	0.011022	0.040319	0.198223	0.017422	0.055623	0.094301	0.143993	0.055797
FIWC	0.035195	0.026681	0.018779	0.052915	0.020177	0.019916	0.008533	0.02893	0.100518

	p-value (Weekly samples, by paired t-test)								
	W0	W1	W2	W2½	W3	W4	W5	W6	W7
Omp	-	0.021642	0.756126	-	0.029106	0.018537	0.030292	0.028624	0.110451
FIM	-	0.003597	0.048875	-	0.01922	0.029491	0.026148	0.001622	0.00501
CPS	-	0.54379	0.088096	-	0.007341	0.036992	0.003199	0.008801	0.028945
LPS	-	0.016963	0.030292	-	0.004179	0.01632	0.011367	0.005093	0.003333
FIWC	-	0.5	0.824337	-	0.931227	0.966425	0.052479	0.000514	0.135238

Lymphocyte proliferation of the urinary tract infection model: Data for Figure (5A), for the values of the Stimulation Index, Standard Error (SE), and p-values along 7 weeks (W0 till W7) for the Outer Membrane Proteins (Omp), Fimbriae proteins (Fim), Capsular Polysaccharides (CPS), Lipopolysaccharides (LPS), and Formalin Inactivated Whole bacterial Cells (FIWC).

Stimulation Index									
Intra-tracheal	**W0**	**W1**	**W2**	**W2½**	**W3**	**W4**	**W5**	**W6**	**W7**
**OMP**	0	2.45342	0.717473	0.638999	1.089662	0.094168	1.427659	0.780252	-0.01345
**FIM**	0	0.250737	0.743363	0	0	0	0	0	0
**LPS**	0	1.296979	1.26383	0.401939	0.488957	0	0.58737	0.501733	0.485274
**CPS**	0	1.211209	1.481718	0.278107	0.706665	0	0.410322	0	0.095742
**FIWC**	0	1.657486	1.339612	0.313925	0.719988	0.657466	1.214239	0.405734	0.088847

	**SE**								
	**W0**	**W1**	**W2**	**W2½**	**W3**	**W4**	**W5**	**W6**	**W7**
**Omp**	0.094378	0.046251	0.061221	0.079962	0.050709	0.110457	0.064209	0.074692	0.045397
**FIM**	0.034384	0.021377	0.050553	0.057304	0.030335	0.073071	0.01325	0.053515	0.013977
**CPS**	0.050591	0.054111	0.039248	0.136468	0.056559	0.022959	0.041639	0.041107	0.064332
**LPS**	0.006635	0.10469	0.037188	0.004187	0.052367	0.048281	0.024244	0.072032	0.080073
**FIWC**	0.020506	0.02906	0.035082	0.060207	0.021822	0.027336	0.070414	0.063489	0.066665

	**p-value (Weekly samples, by paired t-test)**								
	**W0**	**W1**	**W2**	**W2½**	**W3**	**W4**	**W5**	**W6**	**W7**
**Omp**	-	0.005718	0.014363	-	0.074161	0.01299	0.047112	0.012163	0.11372
**FIM**	-	0.860985	0.072299	-	0.030905	0.038467	0.006006	0.00926	0.035997
**CPS**	-	0.112194	0.5	-	0.012141	0.004273	0.011375	0.043835	0.259288
**LPS**	-	0.081376	0.003556	-	0.051505	0.010669	0	0.013824	0.014884
**FIWC**	-	0.050086	0.5	-	0.112309	0.335747	0.019683	0.008058	0.008801

Lymphocyte proliferation of the respiratory tract infection model: Data for Figure (5B), for the values of the Stimulation Index, Standard Error (SE), and p-values along 7 weeks (W0 till W7) for the Outer Membrane Proteins (Omp), Fimbriae proteins (Fim), Capsular Polysaccharides (CPS), Lipopolysaccharides (LPS), and Formalin Inactivated Whole bacterial Cells (FIWC).

Stimulation Index									
Sepsis	W0	W1	W2	W2½	W3	W4	W5	W6	W7
**OMP**	0	16.7966	2.321049	9.185763	32.24663	27.88802	18.71013	16.92062	11.05599
**FIM**	0	15	0	1.818182	0	0	0	0	0
**LPS**	0	8.343301	0.508373	4.400917	4.366029	2.564294	2.616627	0.956938	2.322568
**CPS**	0	5.487973	1.126624	4.981108	1.055202	1.078242	2.792369	0	1.022947
**FIWC**	0	2.33619	1.327067	6.494025	2.059718	2.594231	8.764169	3.755414	1.280066

	SE								
	W0	W1	W2	W2½	W3	W4	W5	W6	W7
**Omp**	0.029376	0.125949	0.039759	0.014962	0.004958	0.054851	0.044663	0.010837	0.028725
**FIM**	0.061885	0.526812	0.04983	0.102867	0.011226	0.097896	0.050271	0.18547	0.083592
**CPS**	0.095403	0.116388	0.04347	0.150729	0.110016	0.145968	0.082169	0.041002	0.020529
**LPS**	0.0599	0.069107	0.117868	0.11736	0.098256	0.06075	0.04125	0.015498	0.056608
**FIWC**	0.040873	0.045194	0.025388	0.049639	0.017141	0.053278	0.061269	0.066918	0.030744

	p-value (Weekly samples, by paired t-test)								
	W0	W1	W2	W2½	W3	W4	W5	W6	W7
**Omp**	-	0.003001	0.004257	-	0.003561	0.008075	0.539209	0.492888	0.010756
**FIM**	-	0.012163	0.009106	-	0.004615	0.030892	0.015624	0.59219	0.582336
**CPS**	-	0.000721	0.002439	-	0.002458	0.059017	0.5	0.024791	0.04205
**LPS**	-	0.004608	0.004508	-	0.5	0.5	0	0.008058	0.010895
**FIWC**	-	0.088218	0.098562	-	0.039386	0.010789	0.023127	0.015148	0.035121

Lymphocyte proliferation of the sepsis model: Data for Figure (5C), for the values of the Stimulation Index, Standard Error (SE), and p-values along 7 weeks (W0 till W7) for the Outer Membrane Proteins (Omp), Fimbriae proteins (Fim), Capsular Polysaccharides (CPS), Lipopolysaccharides (LPS), and Formalin Inactivated Whole bacterial Cells (FIWC).
