# Supplementary material for: Variable progressive behavior of Klebsiella pneumoniae at different sites of infection
Source: Front Immunol. 2026 Apr 13;17:1775450. doi: 10.3389/fimmu.2026.1775450 (PMC13111035; doi:10.3389/fimmu.2026.1775450)
Supplement: Supplementary file 2 [file Image1.pdf]

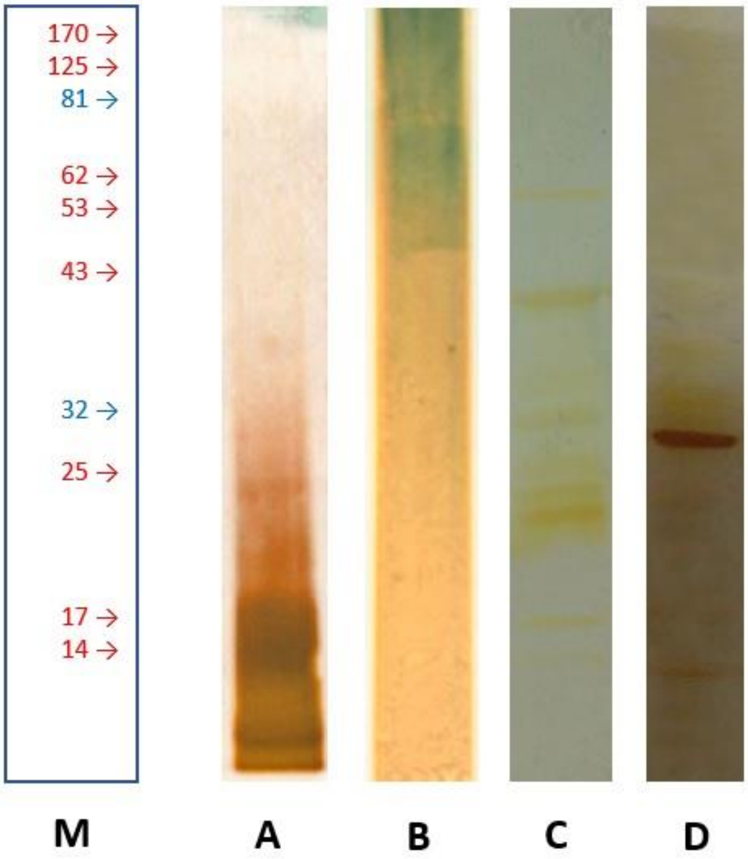

Supplement 1. Panel for the stained SDS-PAGE to show the purity and identity of the tested antigens of *K. pneumoniae*. (A) Purified lipopolysaccharides separated on SDS-PAGE and identified by silver stain then Alcian blue stain. It displays the identity of the LPS, with no sharp bands and no blue stain. (B) Purified capsular polysaccharides separated on SDS-PAGE and stained with by silver stain then Alcian blue stain. It shows one high molecular weight smear stained both with the yellowish silver stain and blue stain, with no sharp bands. (C) Purified outer membrane protein separated on SDS-PAGE and treated by silver stain. The stain shows the typical sharp protein bands of *K. pneumoniae* at molecular weights 15, 17, 20, 32, 40, and 60, while not showing any smear for polysaccharides. (D) Purified fimbriae proteins stained by silver stain, showing a sharp distinct band at 28.8 kDa and bands' zone between 11 and 13 kDa, with no smear. (M) Nippon molecular weight marker chart.
