# Supplementary material for: Variable progressive behavior of Klebsiella pneumoniae at different sites of infection
Source: Front Immunol. 2026 Apr 13;17:1775450. doi: 10.3389/fimmu.2026.1775450 (PMC13111035; doi:10.3389/fimmu.2026.1775450)
Supplement: Supplementary file 3 [file Image2.pdf]

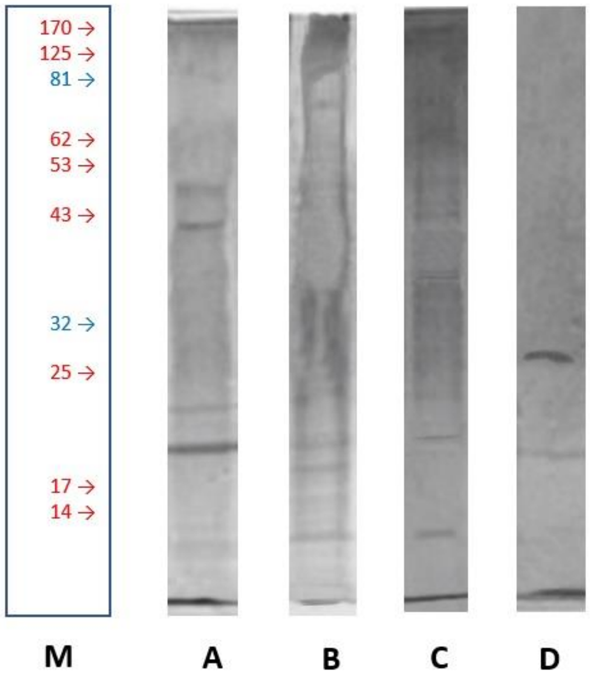

Supplement 2. Representative western blot plates for the raised antisera of the experimental animal models against *K. pneumoniae* components. (A) Antigen-antibody interaction detected at day-3 post-initial infection in SP-model against the OMP sharp-bands. (B) Antigen-antibody interaction detected at day-16 post-initial infection in SP-model against both sharp-bands of OMP, and LPS smear. (C) Antigen-antibody interaction detected at day-49 post-initial infection in SP-model against both sharp-bands of OMP, and LPS smear. (D) Antigen-antibody interaction detected at day-3 post-initial infection in UT-model against sharp-bands of both OMP protein at 20 kDa, and fimbriae protein at 28.8 kDa.
