# Supplementary material for: Variable progressive behavior of Klebsiella pneumoniae at different sites of infection
Source: Front Immunol. 2026 Apr 13;17:1775450. doi: 10.3389/fimmu.2026.1775450 (PMC13111035; doi:10.3389/fimmu.2026.1775450)
Supplement: Supplementary file 4 [file Presentation1.pdf]

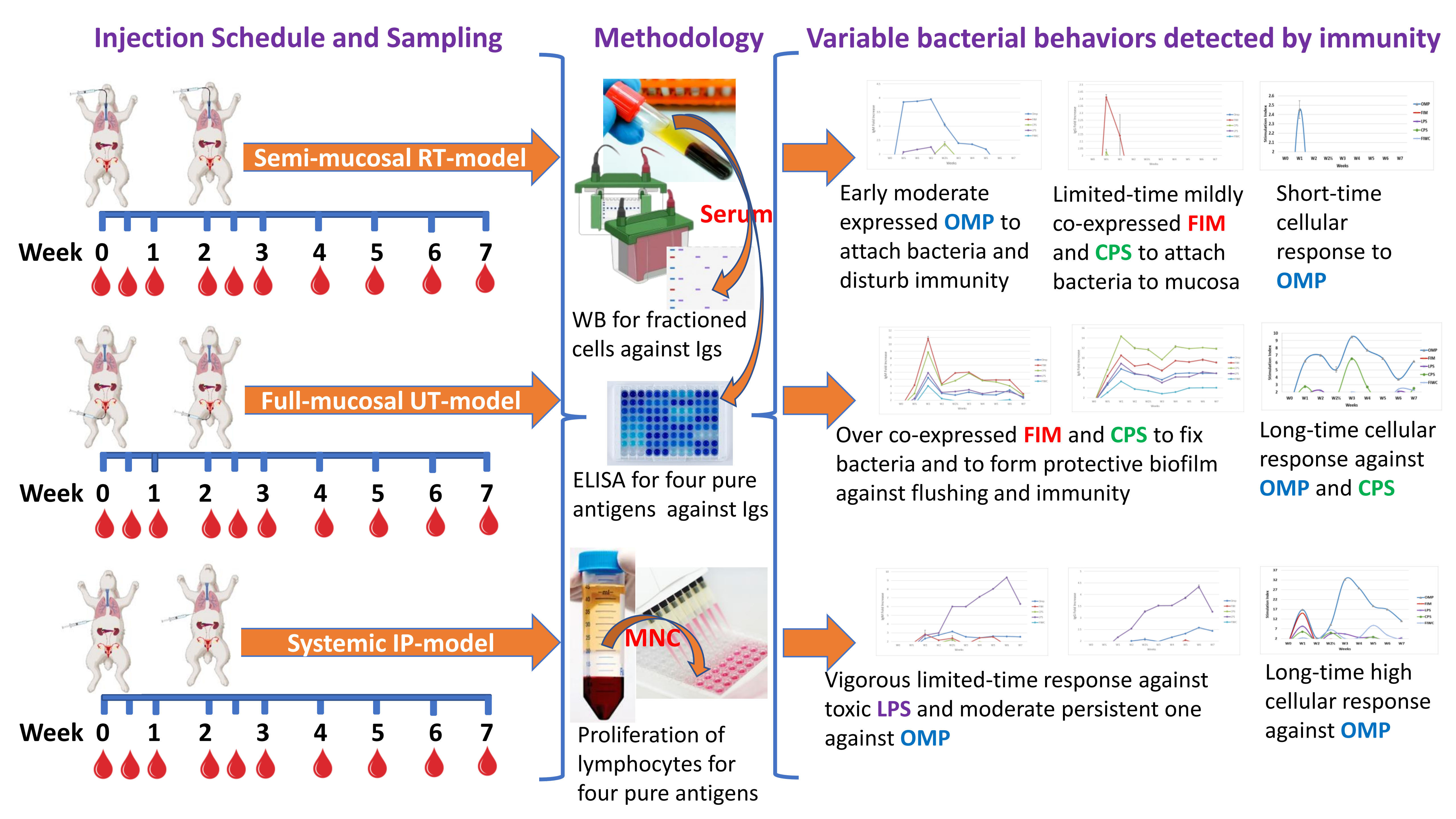

Injection Schedule and Sampling
Methodology
Variable bacterial behaviors detected by immunity
Semi-mucosal RT-model
Week 0 1 2 3 4 5 6 7
Full-mucosal UT-model
Week 0 1 2 3 4 5 6 7
Systemic IP-model
Week 0 1 2 3 4 5 6 7
Serum
WB for fractioned cells against Igs
ELISA for four pure antigens against Igs
MNC
Proliferation of lymphocytes for four pure antigens
Early moderate expressed OMP to attach bacteria and disturb immunity
Limited-time mildly co-expressed FIM and CPS to attach bacteria to mucosa
Short-time cellular response to OMP
Over co-expressed FIM and CPS to fix bacteria and to form protective biofilm against flushing and immunity
Long-time cellular response against OMP and CPS
Vigorous limited-time response against toxic LPS and moderate persistent one against OMP
Long-time high cellular response against OMP
